# HLA‐DRB1 Allelic Combinations Differentially Shape Dendritic Cell Antigen Presentation Enhanced by Tumour Cell Line Lysate‐Pulsing

**DOI:** 10.1111/tan.70563

**Published:** 2026-01-28

**Authors:** Gonzalo Lázaro, Juan A. Cedano, Maitane Faus, Carme Roura‐Mir, Laia Garrigós, José Pérez‐García, Javier Román‐García, Javier Cortés, Andrea Aran, Mercè Martí

**Affiliations:** ^1^ Immunology Unit, Department of Cell Biology, Physiology and Immunology Institut de Biotecnologia i Biomedicina (IBB), Universitat Autònoma de Barcelona (UAB) Bellaterra Spain; ^2^ Department of Biochemistry and Molecular Biology Institut de Biotecnologia i Biomedicina (IBB), Universitat Autònoma de Barcelona (UAB) Bellaterra Spain; ^3^ Fundació de Recerca Clínic Barcelona‐Institut d'Investigacions Biomèdiques August Pi i Sunyer (FRCB‐IDIBAPS) Barcelona Spain; ^4^ International Breast Cancer Center (IBCC), Pangaea Oncology, Quironsalud Group Barcelona Spain; ^5^ Medical Scientia Innovation Research (MedSIR) Barcelona Spain; ^6^ Medical Oncology Department IOB Institute of Oncology‐Madrid Madrid Spain; ^7^ Department of Medicine, Faculty of Biomedical and Health Sciences Universidad Europea de Madrid Madrid Spain; ^8^ Biosensing and Bioanalysis Group Institut de Biotecnologia i Biomedicina (IBB), Universitat Autònoma de Barcelona (UAB) Bellaterra Spain

**Keywords:** antigen presentation, CD4+ T cells, dendritic cells, heterozygosity, HLA‐DRB1 alleles, peptides

## Abstract

The anti‐tumour immune response plays a pivotal role in eliminating tumour cells, with the presence of tumour‐infiltrating lymphocytes (TILs) often correlated with improved patient outcomes. Among these, CD4+ T lymphocytes act as key orchestrators of the immune response, functioning as effector and regulatory cells, and are essential for establishing immunological memory. To better understand the role of CD4+ T cells in anti‐tumour immunity, we analysed the HLA‐II immunopeptidome of dendritic cells (DCs) from HLA‐heterozygous donors pulsed with a protein extract from the MCF‐7 tumour cell line. Our objective was to identify differences in the arrays of peptides binding distinct HLA‐DRB1 allele combinations and the effect of DC pulsing on peptide presentation. We found that presented peptide repertoires are strongly influenced by HLA‐DR heterozygosity in an allele‐specific manner. Alleles with high binding strength (e.g., DRB1*01:01, DRB1*03:01 and DRB1*04:04) tended to dominate peptide presentation; however, this dominance is significantly modulated by the allelic combination, suggesting that antigen presentation is shaped not only by individual allele properties but also by their combinations. Pulsing DCs with MCF‐7 extracts increased peptide overlap between donors and enabled the identification of 58 proteins putatively derived from the tumour cell line lysates. Interestingly, peptide presentation from these proteins reinforced allele‐specific features of dominance and weakness previously observed across the entire immunopeptidome. Gaining insights into the peptide repertoire presented by distinct HLA‐DR combinations could inform the design of personalised immunotherapies based on peptide‐pulsed DCs, ultimately enhancing CD4+ TIL responses across diverse patient populations.

## 1 Introduction

Adaptive immunity plays a crucial role in the immune response against tumours [1]. However, the selective pressure exerted on tumour cells can induce immune evasion through various mechanisms [2]. Against this evasion, cancer immunotherapy aims to stimulate tumour‐specific T cell responses, hereby establishing efficient and long‐lasting antitumor immunity. CD8+ cytotoxic T lymphocytes (CTLs) have traditionally been favoured for adoptive cell transfer due to their ability to target and eliminate tumour cells presenting intracellular antigens via MHC class I molecules (MHC‐I). Nevertheless, CD4+ T cells orchestrate the immune response and are required for the development of immunological memory [3]. They assume a supportive role by primarily recognising extracellular tumour antigens presented by MHC class II molecules (MHC‐II) and contribute to the activation of various immune cells, including CTLs [4, 5].

T cell activation depends on the specific interaction between the T cell receptor (TCR) expressed by CD8+ and CD4+ T cells and peptides complexed with MHC‐I and MHC‐II molecules, respectively [6]. Given the epithelial aetiology of carcinomas, the presentation of tumour peptides by MHC‐II depends on antigen‐presenting cells (APCs), particularly dendritic cells (DCs), which engage in phagocytosis of apoptotic tumour cells or macropinocytosis of secreted tumour proteins [7]. Indeed, a high proportion of these cells within tumours has been shown to correlate with improved prognosis [8]. In cancer research, the MHC‐II immunopeptidome has not been extensively explored because MHC‐I molecules play a more direct role in CTL‐mediated tumour cell killing. However, a thorough understanding of the MHC‐II peptide repertoire is essential for comprehensively characterising CD4+ T cells and cellular immune dynamics [9] in anti‐tumour responses.

Proteins present in the extracellular space and the cell membrane are the primary sources of peptides within the endocytic compartments, where proteases such as cathepsins degrade these proteins, generating peptides for presentation by MHC‐II [10]. However, intracellular proteins can also contribute to the MHC‐II peptide pool through biological processes such as autophagy, which allows cytosolic proteins to access the endocytic pathway [11]. The HLA‐DM chaperone is required for stabilising MHC‐II heterodimers in endocytic compartments, ultimately facilitating peptide exchange for high‐affinity ligands [10]. Once formed, peptide‐MHC‐II (pMHC‐II) complexes are displayed on the APC's outer membrane. Additionally, the presence of danger signals or immunosuppression within the tumour microenvironment (TME) can enhance or diminish, respectively, this physiological process [12, 13]. Under such conditions, peptides originating from intracellular proteins of stressed or damaged cells may be released into the extracellular space, potentially displacing the homeostatic immunopeptidome of MHC‐II+ cells. This shift may help to overcome CD4+ T cell tolerance [12, 14].

The MHC (HLA in humans) is the most polymorphic mammalian genetic system [15]. HLA‐II proteins encompass HLA‐DR, HLA‐DP and HLA‐DQ, all of which share the same quaternary structure [16]. HLA‐DR molecules consist of two chains: the minimally polymorphic DRA‐encoded α chain and the β chain encoded by the highly polymorphic HLA‐DRB gene complex with over 2000 variants identified [17]. Unlike MHC‐I, the antigen‐binding groove in MHC‐II molecules is formed by the α1 and β1 domains, creating a wide structure that can accommodate long peptides typically ranging from 13 to 25 amino acids. Although the class II peptide‐binding core is also typically a 9‐mer, the presence of peptide flanking residues (PFRs) enhances the versatility of peptide presentation [18, 19].

In this complex landscape, the study of the MHC‐II immunopeptidome is particularly challenging. In addition to general characteristics of the MHC system, such as (i) the HLA polymorphism [15], which primarily affects the antigen‐binding site and imposes allele‐dependent restrictions that result in allele‐specific peptide repertoires and (ii) the strong linkage disequilibrium among HLA‐II genes [20], several MHC‐II‐specific features further contribute to this complexity. These include (iii) the variable peptide length and the presence of peptide flanking regions (PFRs) that influence peptide presentation [21], (iv) the highly variable affinity of different HLA‐DR alleles for the invariant chain‐derived CLIP peptide [22] and (v) the critical role of HLA‐DM in peptide editing and loading, which creates a competitive environment for peptides generated through endosomal degradation [23]. Consequently, in silico HLA‐II affinity prediction methods still exhibit lower performance compared to their HLA‐I counterparts [24].

Studies of class II immunopeptidome have mostly relied on HLA homozygous cell lines to precisely define binding motifs for different alleles. Thus, research exploring the peptide repertoire from heterozygous individuals, the most common genetic state in humans, remains limited [10]. However, the immunopeptidome presented by a specific HLA‐II allele might be influenced by its co‐expressed allele in heterozygous individuals. While heterozygosity may broaden target protein coverage [25], the presentation of specific antigens remains constrained by the individual's haplotype. HLA class I heterozygosity has been shown to influence cancer risk in large cohorts [26] and the response to immunotherapy for melanoma and lung cancer [27], while heterozygosity for both HLA‐I and ‐II has been linked to a reduced risk of colorectal cancer [28]. However, the mutual influence between co‐expressed alleles for each locus has not been studied at the population level. Interestingly, a recent study clustering HLA‐II binding patterns suggests that heterozygosity does not always enhance antigen diversity [29].

To investigate the impact of HLA‐II heterozygosity on the presentation of tumour‐associated antigens, we randomly selected 14 donors to generate monocyte‐derived dendritic cells (MoDCs). Six samples were pulsed with lysates from the MCF‐7 breast cancer cell line, while eight samples were used as controls. Following high‐resolution HLA typing, we conducted a comparative analysis to (i) identify differences in peptide‐binding patterns among various HLA‐DRB1 allele combinations, (ii) evaluate whether allele co‐expression influences the presentation of potential tumour‐related peptides identified upon pulsing. Our findings indicate that specific HLA‐DR allele co‐expression shapes the composition and dominance hierarchy of the tumour‐derived immunopeptidome, reinforcing allele‐specific patterns of peptide selection upon antigen exposure.

## 2 Material and Methods

### 2.1 Obtention of MCF‐7 Cell Lysates

MCF‐7 human breast cancer cell line (luminal A, oestrogen and progesterone receptor‐positive [30]) was cultured at 3 × 10^5^ cells/ml in DMEM GlutaMAX, 10% decomplemented foetal bovine serum (FBS) (Gibco) and 1% Penicillin–Streptomycin (P/S) (Sigma‐Aldrich), at 37°C and 5% CO_2_. Dry pellets containing 5 × 10^6^ cells were collected to obtain protein extracts. Cells were lysed by exposing them to 10 freeze–thaw cycles, followed by a 2‐h incubation with rotation at 4°C in 1 mL lysis buffer (50 mM Tris‐HCl at pH 8.0, 150 mM NaCl). After 10 min centrifugation at 12,000 × *g* and 4°C to remove cell debris, supernatants were stored for later use in MoDC pulsing. Five different MCF‐7 lysates were quantified, resulting in 903 ± 60.3 μg of total protein. A small fraction of these lysates was stored at −80°C for analysing the MCF‐7 proteome.

### 2.2 Protein Digestion for Proteome Analysis

Stored MCF‐7 protein extracts as described above were digested using Sequencing Grade Modified Trypsin (Promega) following the FASP (filter aided sample preparation) digestion protocol [31]. The resulting tryptic peptide mixture was then evaporated and desalted using C18 tips (ZipTip Pipette Tips C18, p10 from Merck Millipore) prior to MS analysis.

### 2.3 Proteome Analysis: Tryptic Peptide Identification and Analysis by Mass Spectrometry (MS)

Reconstituted peptides were analysed using an Orbitrap Fusion Lumos Tribrid mass spectrometer (Thermo Fisher) equipped with a Thermo Scientific Dionex Ultimate 3000 ultrahigh‐pressure chromatographic system (Thermo Fisher) and Advion TriVersa NanoMate (Advion Biosciencies) as the nanospray interface. Peptides mixtures were loaded to a μ‐precolumn Acclain C18 PepMap100 Trap column (100 μm × 2 cm, 5 μm, 100 Å, C18 Trap column; Thermo Fisher) at a flow rate of 15 L/min and separated using a C18 Acclaim PepMap RSLC analytical column (75 μm, 50 cm, 2 μm, 100 Å, nanoViper). Separations were done at 200 nL/min using a linear acetonitrile (ACN) gradient from 0% to 40% B in 240 min (solvent A = 0.1% formic acid (FA) in water, solvent B = 0.1% FA in ACN). The mass spectrometer was set up in the positive ion mode and the analysis was performed in an automatic data‐dependent mode (DDA) (a full scan followed by 10 HCD scans for the most abundant signals).

### 2.4 Blood Samples and Generation of Mature MoDCs

Leukocyte residues from 14 healthy donors were obtained from the Biobanco del Centro de Hemoterapia y Hemodonación (Valladolid, Spain). PBMCs were isolated from leukocyte residues by separation over a Ficoll‐Paque PLUS density gradient (Lymphoprep, Alere Technologies). Monocytes were isolated by adherence after a 2‐h incubation at 37°C and 5% CO_2_, then differentiated into immature MoDCs by culture at 1 × 10^6^ cells/ml with IMDM GlutaMAX (Gibco), with 1% P/S and 5% decomplemented human serum (in‐house prepared), in the presence of GM‐CSF (450 U/mL) and IL‐4 (300 U/mL). On day 3, immature MoDCs were harvested and re‐seeded at a concentration of 1 × 10^6^ cells/ml in 10 mL of fresh medium supplemented with renewed cytokines. After an additional 3 days, MoDCs were matured after 6 days, adding a cytokine maturation cocktail (TNF‐α (500 U/mL), IL‐1β (1000 U/mL), IL‐6 (1000 U/mL) and prostaglandin E2 (PGE‐2) (10 μg/mL)) for 24 h.

### 2.5 Phenotype Characterisation of MoDCs

MoDCs were stained with anti‐human antibodies anti‐CD3 PerCP, anti‐HLA‐ABC APC, anti‐HLA‐DR FITC (BD Pharmingen), anti‐CD80 PE, anti‐CD83 PE and anti‐CD86 FITC (Milteny Biotec), for 20 min at 4°C in the dark diluted in PBS containing 2% FBS following the manufacturer's recommendations. Cells were then washed twice, collected in 0.2 mL PBS, and analysed by flow cytometry using a BD FACS Canto cytometer. Analyses were performed using the FlowJo and FACS Diva software.

### 2.6 Pulsing of MoDCs With MCF‐7 Protein Extracts

Immature MoDCs exhibiting high viability were exposed to a maturation cocktail and simultaneously subjected to antigen pulsing for 24 h, using the complete extract obtained from 5 × 10^6^ MCF‐7 cells, corresponding to a final protein pulsing concentration of 90 μg/mL in the culture medium. Non‐pulsed MoDCs were also included as controls for subsequent peptide analysis. Following incubation, both pulsed and non‐pulsed mature MoDCs were harvested, and dry pellets were collected for the purification of HLA‐bound peptides.

### 2.7 HLA Typing of Modcs' Donors

Lymphocytes discarded after monocyte isolation were used for all donors' HLA typing, conducted at the Banc de Sang i Teixits (Barcelona, Spain) and following standard procedure. The QIAsymphony DNA kit (Qiagen) was used for genomic DNA extraction following the manufacturer's instructions. Genomic DNA was genotyped for HLA class I and class II molecules (HLA‐A, ‐B, ‐C, ‐DRB1, ‐DQB1, ‐DPB1 genes) at high resolution. Six loci were genotyped simultaneously by an in‐house multiplex long‐range PCR (LRPCR). The library was prepared (enzymatic fragmentation, adapter ligation and barcoding) from the PCR pools using the NGSgo full kit (GenDx, Utrecht, The Netherlands) according to the manufacturer's instructions. The final denatured library was sequenced using a MiSeq sequencer (Illumina, San Diego, CA, USA). HLA class I and II genotype determination was performed with NGSengine 3.3.0 software (GenDx) using the IMGT/HLA updated database as a reference.

### 2.8 Purification of HLA‐DR Complexes

Peptide‐HLA‐DR complexes were purified from pulsed and non‐pulsed MoDC samples, using standard procedure. Briefly, cell pellets were resuspended in 0.5 mL of solubilisation buffer (lysis buffer + 10 mg/mL n‐dodecyl β‐D‐maltoside), sonicated four times (4×) for 5 s (20% amplitude of maximum 130 W), and incubated in rotation at 4°C for 2 h. The solubilised membranes were sonicated 4× under the same conditions and finally centrifuged at 4°C for 1 h at 20,000 × *g*. HLA‐DR‐peptide complexes in the supernatant were collected from the soluble fraction and incubated in rotation at 4°C for 18 h with 0.01 mL of CNBr‐activated sepharose beads (GE Healthcare Life Sciences) coupled to the monoclonal anti‐HLA‐DR antibody (clone L243, Bio X Cell). Prior to incubation, the beads were washed with three volumes of lysis buffer. After incubation, HLA‐DR‐peptide complexes were purified by immunoprecipitation using Bio‐Spin Chromatography Columns (Bio‐Rad). Non‐retained fractions were collected and stored for future analysis. Retained fractions were washed 3× with 50 mM Tris‐HCl (pH 8.0), 150 mM NaCl, and 0.5% n‐dodecyl β‐D‐maltoside, and then 3× with 50 mM Tris‐HCl (pH 8.0) and 150 mM NaCl. Unspecific interactions were reduced by washing with a high‐salt concentration buffer (50 mM Tris‐HCl at pH 8.0, 0.5 M NaCl). HLA‐DR‐peptide complexes were eluted with a 0.25% TFA solution after washing 3× with 50 mM Tris‐HCl (pH 8.0) and 150 mM NaCl and 1× with 20 mM Tris‐HCl (pH 8.0). The eluted complexes were desalted and cleared using C18 tips and strong cation exchange (SCX) based analytical methods (TopTips with PolySulfoethyl A from Cluzeau) before MS analysis.

### 2.9 HLA‐DR Repertoires From MoDCs. Peptide Identification by MS

Eluted peptides were analysed by liquid chromatography coupled to high‐resolution mass spectrometry (LC‐HRMS) using two comparable but independently optimised workflows. In the first workflow, peptides reconstituted in 6 μL of 0.1% FA were analysed by LC‐HRMS on an LTQ Orbitrap XL mass spectrometer (Thermo Fisher) equipped with a nanoESI source and coupled to an Agilent 1100 nanochromatographic system, using a 140‐min ACN gradient. Data were acquired in DDA mode at 60,000 resolution (m/z 400), with fragmentation of the 10 most intense peaks via CID and dynamic exclusion of 45 s. Raw data were processed in Proteome Discoverer 1.4 (UniProt/SwissProt) with the following parameters: no enzyme, 20 ppm precursor mass tolerance, 0.02 Da fragment tolerance and variable modification of oxidised methionine (+16 Da). Peptide spectral matches (PSMs) were filtered at 1% FDR using Percolator. In the second workflow, peptides were reconstituted in 5 μL of 0.1% FA and analysed on an Orbitrap Exploris 240 mass spectrometer (Thermo Fisher) coupled to an Ultimate 3000 nanoHPLC system via an EASY‐Spray ion source. Chromatographic separation was performed using a PepMap Neo C18 trapping column (300 μm × 5 mm; Thermo Fisher) and an analytical PepMap RSLC c18 column (75 μm × 50 cm, Thermo Fisher), with solvent A (0.1% FA) and solvent B (0.1% FA, 80% ACN). Peptides were separated at a flow rate of 250 nL/min at 50°C under the following gradient elution: 4% B for 2 min, linear increase to 35% B in 63 min, linear increase to 50% B in 6 min, linear increase to 90% B in 4 min, 90% B for 1 min, linear decrease to 4% B in 1 min and 4% B for 10 min. Data were acquired in DDA mode, with each acquisition cycle consisting of one survey scan (355–1200 m/z) at 60,000 resolution (FWHM) and up to 25 MS/MS scans at 15,000 resolutions (FWHM). Precursors with charge states +1 to +5 were selected for fragmentation at 30% HCD collision energy using a dynamic exclusion window of 25 s. Raw MS/MS data were searched with Peaks Studio 7.5 (Bioinformatics Solutions) against the Human Reference Proteome (20,359 canonical entries), using the following parameters: non‐specific cleavage, MS tolerance of 10 ppm, MS/MS tolerance of 0.02 Da and oxidation of M and pyroglutamic acid formation from N‐terminal Q as variable modifications. Peptide identifications were filtered at < 1% FDR.

### 2.10 HLA‐DR Peptide Repertoire Analysis

HLA‐DR‐bound peptides identified in 3.9 were selected after exclusion of (i) keratin‐derived peptides as possible handling contaminants; (ii) bovine‐derived peptides from FBS from cell culture and (iii) peptides with a length out of the 9–25 aa range, established for HLA class II peptides. The UniProt [32] database was used to assign the specific subcellular localisation of the corresponding parental proteins (peptide‐generating proteins, hereafter referred to as PGPs). PGPs from more than one subcellular localisation were counted in both localisations. The affinity of the selected peptides to the HLA‐DRB1 alleles corresponding to each sample was assessed using the NetMHCIIpan‐4.1. tool [33]. Peptides considered strong binders (SB) (% Rank < 1%) or weak binders (WB) (1% < % Rank < 5%) were maintained in the analysis. Allele assignment for the identified peptides was confirmed using Gibbs clustering [34]. Peptide and PGP overlap between samples was analysed manually using spreadsheets and pivot tables. Upset plots were generated using R (version 4.3.1) and the packages ‘dplyr’, ‘ggplot2’, ‘RColorBrewer’, ‘UpSetR’, ‘Pheatmap’ and ‘Circlize’. Some data were also plotted using GraphPad Prism 8.0.

### 2.11 Multidimensional Scaling (MDS)

All peptide sequences, regardless of their allele origin, were globally aligned using the BLOSUM62 matrix. This alignment enabled the grouping of peptides with high sequence homology, thereby reducing analysis dimensionality by considering each group as a single dimension. The resulting peptide groups were used to position alleles in a multidimensional space. We then applied MDS to project the relationships between alleles into a two‐dimensional (2D) space, allowing visualisation of allele similarity based on their associated peptide repertoires. To assess the impact of heterozygosity, we compared the results obtained from both non‐filtered and filtered datasets. In the non‐filtered dataset, all peptides were included regardless of their binding affinity distribution. In contrast, the filtered approach assigned each peptide—when predicted to bind both alleles of a heterozygous sample—exclusively to the allele with the higher predicted binding affinity. This strategy minimised peptide overlap between alleles with similar binding motifs, thereby improving the resolution of allele‐specific presentation patterns. For a detailed overview of the computational pipeline, see Figure S1.

## 3 Results

### 3.1 Characterisation of the HLA‐DRB1‐Associated Peptide Repertoire in Heterozygous MoDCs

**FIGURE 1:**
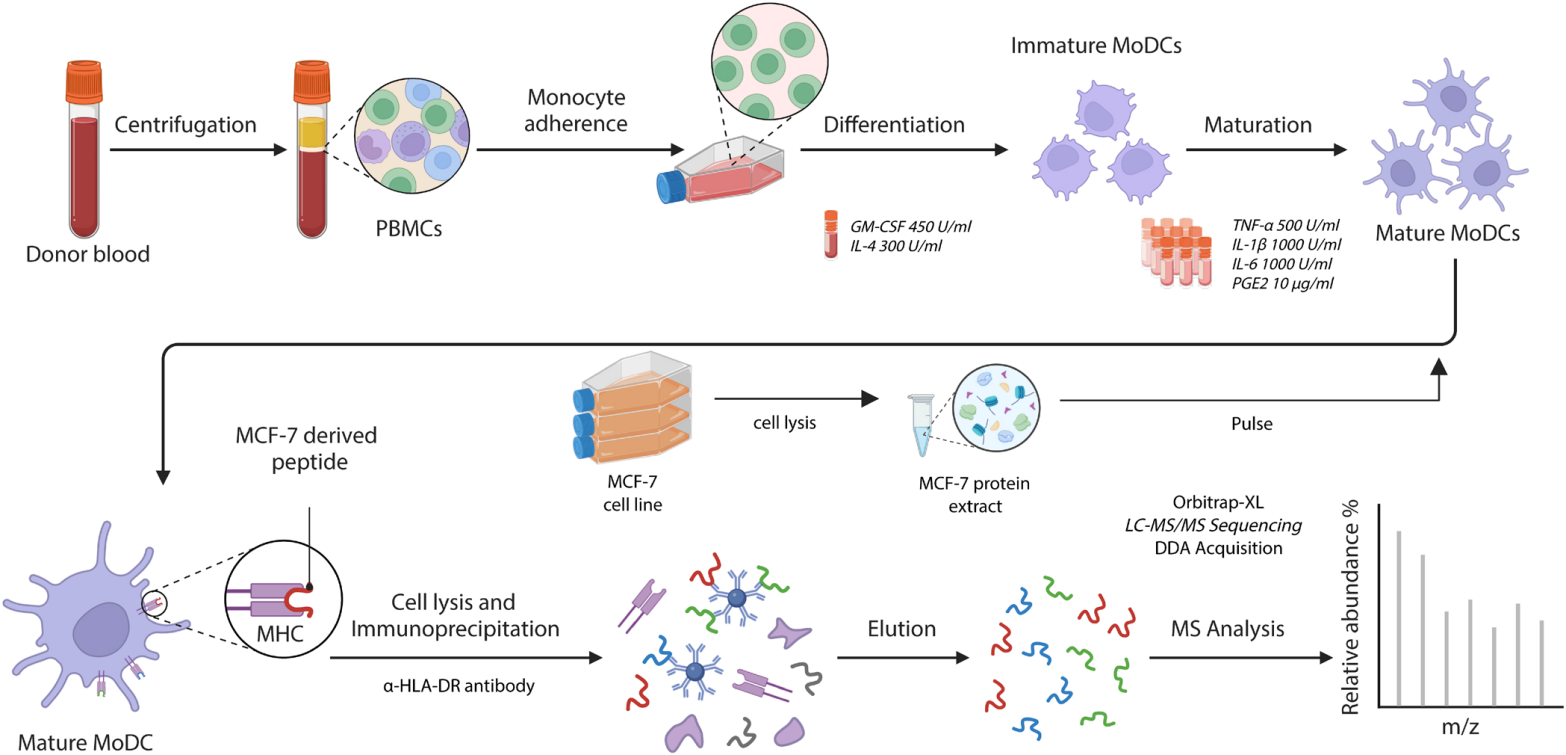
Schematic representation of the experimental procedure carried out to generate MoDCs pulsed with protein extracts derived from the MCF‐7 tumoral cell line. Immunoprecipitations were performed using an α‐HLA‐DR antibody (L243) to isolate MCF‐7‐induced peptides, which were subsequently identified by mass spectrometry. Created with BioRender.com.

Mature MoDCs from 14 random healthy donors of different HLA typing were generated (Table S1). The study followed a workflow summarised in Figure 1, which illustrates the generation of mature, lysate‐pulsed MoDCs and the subsequent purification and MS analysis of HLA‐DR‐derived peptides. Six were pulsed with tumoral cell line MCF‐7 cell lysates while the remaining eight were used as controls. MoDCs were analysed by flow cytometry to confirm the expression of phenotypic markers indicative of activation and maturation, namely HLA‐DR, CD80, CD86 and CD83 (Figure S2). Pulsed and control MoDC preparations (hereafter referred to as P‐DC and C‐DC, respectively) containing 6 ± 3.65 × 10^6^ cells, were used for HLA‐DR immunoprecipitation, yielding a total of 2899 HLA‐DR‐associated peptides (median, IQR: 195, 137–366) derived from 634 PGPs, as detailed in Table S1. The total number of peptides identified per sample did not differ significantly between P‐DC and C‐DC groups (unpaired *t*‐test, *p* = 0.70).

The characterisation of the peptide repertoires obtained from each sample included peptide size distribution and the subcellular localisation of the PGPs. All samples exhibited a normal distribution for MHC‐II‐associated peptides [35, 36] with an average peptide length of 15‐mers (Figure S3A) and included several nested‐set identifications (Table S2). Pulsing with MCF‐7 cell lysates did not result in major differences in the subcellular localisation of the PGPs between P‐DC and C‐DC samples. While most proteins in both sample groups originated from the cell membrane, extracellular matrix and cytosol, P‐DCs showed a moderate increase in the proportion of nuclear (*p* < 0.01) and mitochondrial (*p* < 0.05) proteins, accompanied by a decrease in proteins from the cell membrane and extracellular (*p* < 0.05) compartments. This shift may be attributed to the removal of partially solubilised cellular membranes by centrifugation during MCF‐7 extract preparation, potentially leading to a decrease in secreted and membrane‐derived proteins in P‐DCs (Figure S3B).

Predicted binding to either one or both HLA‐DR alleles was determined for all peptides. After removing contaminants, 2863 peptides derived from 627 PGPs were available for the HLA‐DRB1 affinity analysis. Peptides with no affinity for either of the two alleles in the corresponding sample were excluded, resulting in 1947 peptides derived from 436 PGPs, with a predicted weak (WB) or strong (SB) binding affinity (Table S1) by NetMHCIIPan 4.1. The normalised fraction of SB and WB peptides was calculated for each sample, revealing that certain alleles (e.g., HLA‐DRB1*01:01, HLA‐DRB1*01:02 and HLA‐DRB1*04:04) displayed a stronger affinity for binding peptides compared to others (e.g., HLA‐DRB1*11:01, HLA‐DRB1*13:01, HLA‐DRB1*13:02 and HLA‐DRB1*13:05) (Figure S4). Hereafter, HLA‐DRB1* will be abbreviated as DRB1*. Similarly, the samples C‐DCX and P‐DCX will be referred to as C‐X and P‐X, respectively.

### 3.2 HLA‐DRB1 Allele Dominance Is Shaped by Heterozygosity

**FIGURE 2:**
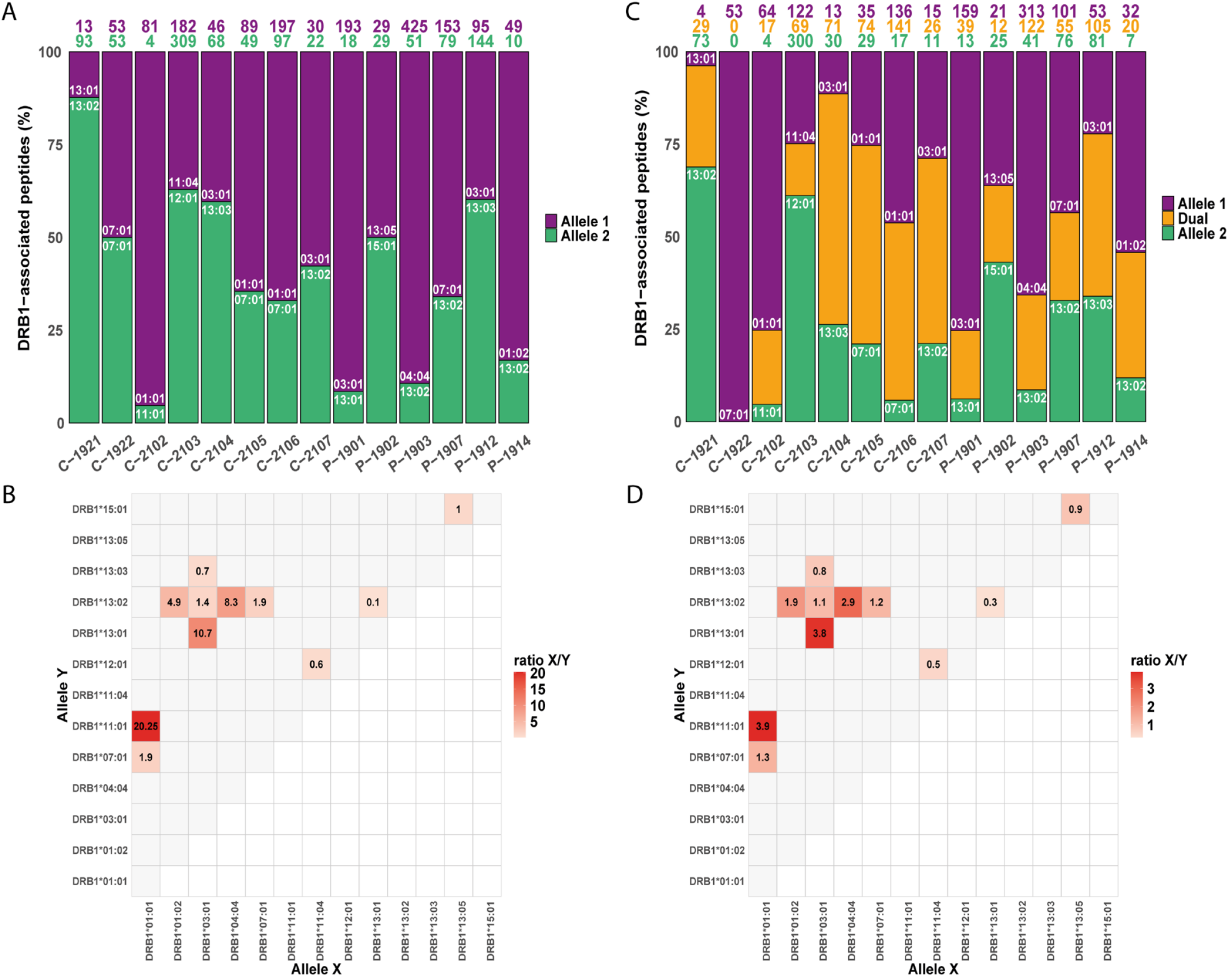
Distribution and binding of peptides across different samples and allele combinations. (A) Percentage of peptides associated with each HLA‐DRB1 allele in MoDC samples, including non‐pulsed (C‐) and pulsed (P‐) samples. Coloured numbers on top of the bars represent the number of peptides corresponding to each percentage. The colour denote the categories: Purple = allele 1, green = allele 2, orange = dual peptides. In some samples, for example, C‐1921, C‐2102, P‐1901, P‐1903 and P‐1914, there was a bias in the allelic distribution of peptides, while in others it was balanced. (B) Ratios of the number of peptides exclusively associated with Allele X (*x*‐axis) versus Allele Y (*y*‐axis), calculated for all MoDC samples. The ratio varies depending on the combination of alleles; for example, DRB1*01:01 and DRB1*03:01 show high ratio values when co‐expressed with some alleles (e.g., DRB1*11:01 and DRB1*13:01, respectively) but lower ratios with others (e.g., DRB1*07:01 and DRB1*13:02 or DRB1*13:03, respectively). (C) The same distribution as in (A) but including dual peptides in every sample. The proportion of dual peptides also varied significantly, ranging from more than 50% in some samples, for example, C‐2104, C‐2105 and P‐1912, to less than 20% in others, for example, C‐1921 and C‐1921. (D) X/Y ratios considering dual peptides in both co‐expressed alleles. Although allele dominance was generally reduced, certain alleles, for example, DRB1*01:01 in C‐2102, DRB1*03:01 in P‐1901, DRB1*04:04 in P‐1903 and DRB1*13:02 in C‐1921, still displayed dominant behaviour over its co‐expressed alleles.

To evaluate the influence of heterozygosity on the arrays of peptides potentially available for presentation, peptides in every sample were assigned to each allele regardless of prior MoDC pulsing. In this context, dominance denotes a relative skew in peptide repertoire composition and binding preferences between co‐expressed HLA‐DRB1 alleles in heterozygous samples. A variable proportion of dual peptides (i.e., peptides attributable to both alleles in a given sample) was identified in all samples, ranging from 14% to 62% (Table S1). This affinity distribution analysis was performed under two conditions. In the first condition (hereafter referred to as ‘filtered’), each peptide was assigned to the allele with the highest predicted binding affinity score (i.e., one peptide per allele; Figure 2A,B). In the second condition (hereafter referred to as ‘non‐filtered’), dual peptides were assigned to both co‐expressed alleles, regardless of differences in predicted binding strength (i.e., each dual peptide assigned to both alleles; Figure 2C,D).

The distribution of filtered‐peptides revealed a predominance in peptide binding of alleles in some samples (e.g., DRB1*01:01 in C‐2102, DRB1*01:02 in P‐1914, DRB1*03:01 in P‐1901, DRB1*04:04 in P‐1903 and DRB1*13:02 in C‐1921). This contrasted with the arrays of peptides obtained from other samples that exhibited a more balanced distribution of peptides between co‐expressed alleles (Figure 2A). Then, the ratio of filtered peptides assigned to allele X versus allele Y (ratio X/Y) was calculated (Figure 2B) as a measure for prevalence of peptides presented by allele X versus allele Y. A ratio X/Y > 1 indicated dominance of allele X over allele Y in terms of peptide presentation. Among the alleles, DRB1*01:01, DRB1*03:01 and DRB1*04:04 exhibited the highest dominance in peptide binding, although this effect was highly dependent on the combination of alleles. For instance, DRB1*01:01 demonstrated the greatest prevalence when co‐expressed with DRB1*11:01 (ratio 20.25) but was less dominant when co‐expressed with DRB1*07:01 (ratio 1.9). Similarly, DRB1*03:01 showed clear dominance when co‐expressed with DRB1*13:01 (ratio 10.7) but not when co‐expressed with DRB1*13:02 (ratio 1.4) or DRB1*13:03 (ratio 0.7). Conversely, DRB1*13:02, which was co‐expressed with five different alleles, generally exhibited weaker binding strength (1/ratio < 1), except when combined with DRB1*13:01 (1/ratio 10). This analysis revealed that the dominance of certain alleles is dependent on the co‐expressed allele (Figure 2B).

Together, these findings indicate that dominance is not an intrinsic and fixed property of individual HLA alleles but is rather modulated by the specific allelic combinations present in heterozygous individuals. This underscores the importance of accounting for allelic context when interpreting immunopeptidomics profiles and predicting immune responses.

In the non‐filtered distribution analysis, dual peptides were considered to assess whether they interfered with the previously observed dominance associated with certain alleles in specific combinations. Most dual peptides displayed strong binding (SB) to one allele but weak binding (WB) to the co‐expressed allele, or vice versa. This pattern was not observed in samples co‐expressing DRB1*03:01 and DRB1*13:03 (P‐1912 and C‐2104), where no clear bias of SB and WB peptide assignment between the two alleles was observed. Although their inclusion led to a more balanced peptide distribution between alleles, as reflected by DRB1‐specific peptide counts (Figure 2C) and X/Y ratios (Figure 2D), certain alleles still exhibited a preferential presentation compared to their co‐expressed alleles, for example, DRB1*01:01 in C‐2102, DRB1*03:01 in P‐1901, DRB1*04:04 in P‐1903 and DRB1*13:02 in C‐1921 (Figure 2C,D). Given the variable presence of dual peptides in our samples, their inclusion in the analysis was essential to fully assess their impact on allele‐specific dominance.

### 3.3 Including Dual Binder Peptides Reveals Allelic Properties and Dominance Relationships

**FIGURE 3:**
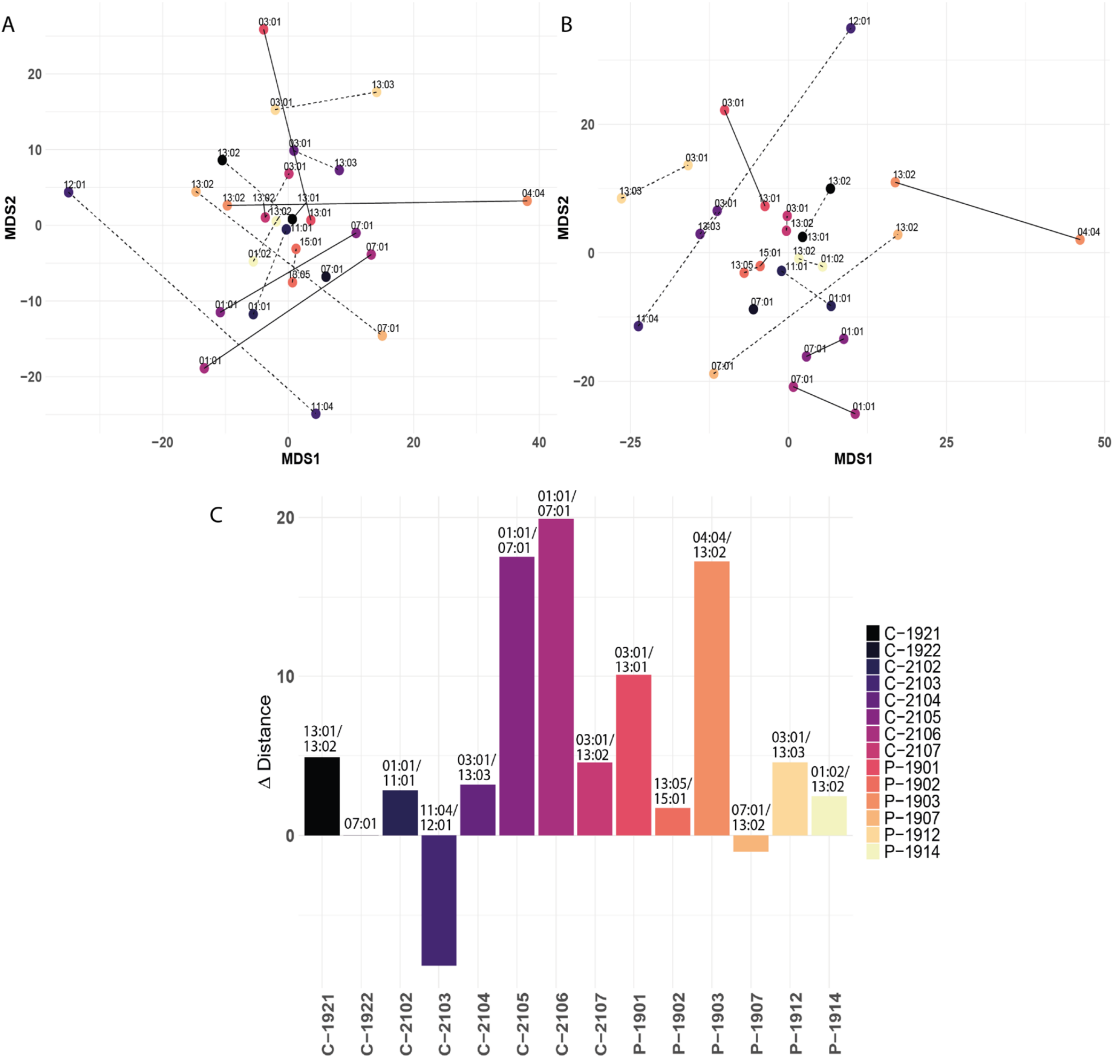
Impact of peptide assignment on HLA‐DRB1 allele clustering in multidimensional scaling (MDS) analysis. MDS was employed to analyse the relationship between HLA‐DRB1 alleles based on antigen presentation patterns. (A) Filtered condition. 2D MDS distribution of HLA‐DRB1 alleles from various donors, clustered by peptide sequence similarity. In heterozygous donors, peptides with positive binding scores for both alleles were assigned exclusively to the allele with the higher affinity score. (B) Non‐filtered condition. The same distribution as in (A) but including all peptides with a positive binding score for each allele. (C) Comparison of allele positions in (A) and (B). Filtering out dual peptides resulted in minimal positional shifts for most co‐expressed alleles, with notable changes observed in certain cases of strong dominance (e.g., DRB1*04:04 over DRB1*13:02) or when both co‐expressed alleles share similar and non‐restrictive peptide‐binding patterns (e.g., DRB1*01:01 and DRB1*07:01). Together, these comparisons highlight the impact of allele competition on peptide presentation and underscore the importance of incorporating binding strength into immunopeptidomic data interpretation.

To confirm whether dual peptides modified the dominance patterns of HLA‐DRB1 alleles, we performed an MDS analysis (see Methods 3.11) under both filtered (Figure 3A) and non‐filtered (Figure 3B) conditions. This analysis visualised the relative distribution of alleles in a 2D space based on their peptide‐binding profiles (see Figure S1). Including dual peptides in the MDS analysis led to a reduction in the relative distance between co‐expressed alleles in the 2D plot, allowing the calculation of distance changes between the two conditions (Figure 3C). This allele convergence was particularly pronounced in: (i) DRB1*04:04/*13:02, where the dominance of DRB1*04:04 dragged DRB1*13:02 much closer. A similar, though less pronounced effect was also observed for DRB1*03:01 when co‐expressed with DRB1*13:01; (ii) DRB1*01:01/*07:01, where both alleles shared similar and minimally restrictive binding patterns, resulting in a comparable shift for both. However, in cases where co‐expressed alleles had distinct and not particularly restrictive binding motifs, such as DRB1*11:04 and DRB1*12:01 in C‐2103, dual peptide numbers were relatively low. Consequently, filtering had little effect on their relative positions. While most allele pairs shifted further apart upon filtering, this particular pair instead moved slightly closer. Given their distinct peptide repertoires and minimal overlap, we hypothesise that this shift reflects an indirect effect of broader redistribution in the 2D space as other pairs adjusted their positions. Overall, the MDS analysis supports the notion that heterozygosity plays a pivotal role in shaping peptide presentation patterns, offering a representation that more accurately reflects physiological conditions in a normal non‐inbred population.

### 3.4 Patterns of Shared Peptide Distribution Are Influenced by Specific HLA‐DR Allele Co‐Expression and by Cell‐Lysate Pulsing

**FIGURE 4:**
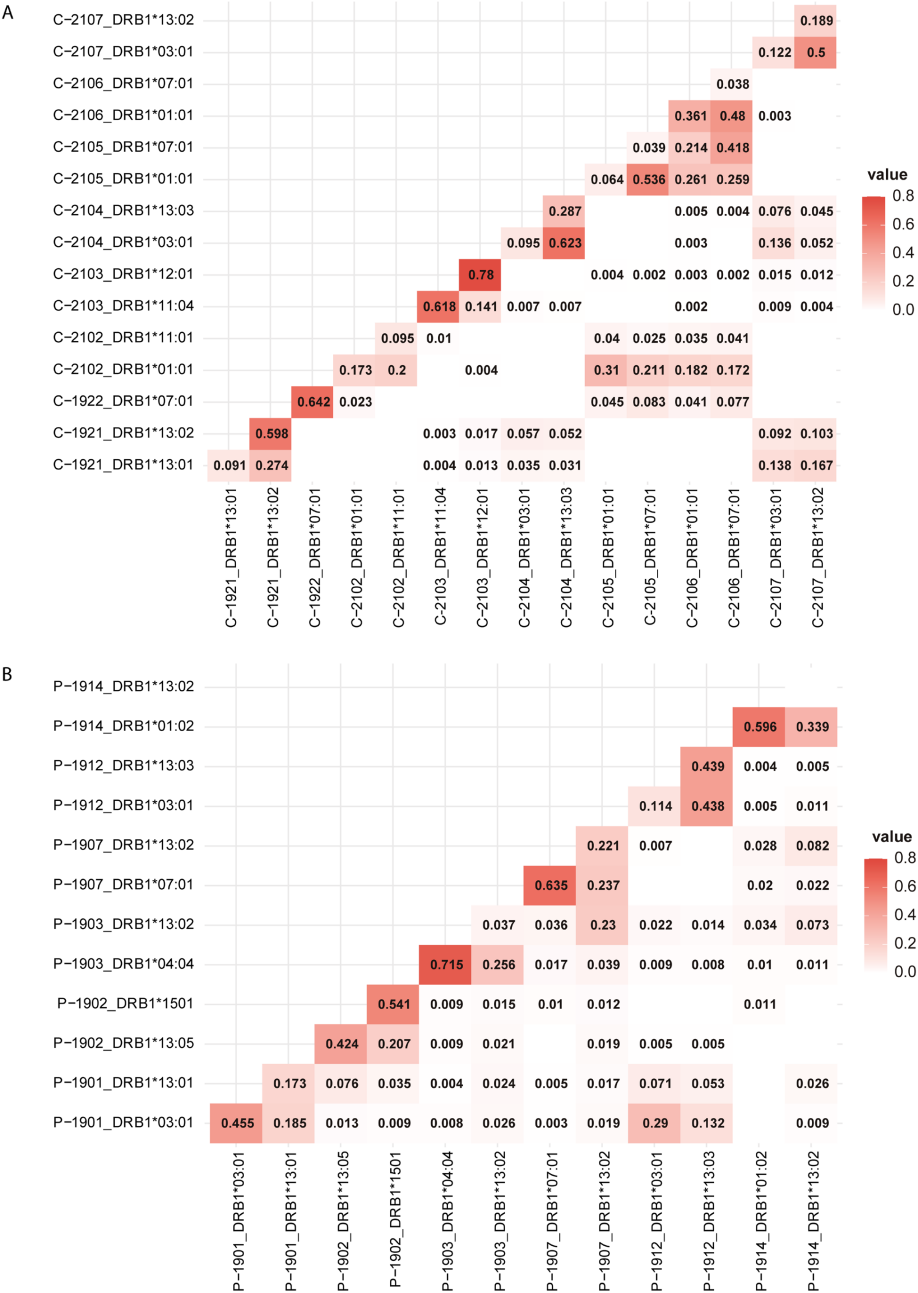
Heatmap showing the frequency of peptide overlap between DC control (A) and pulsed (B) samples and alleles. Diagonal cells display the fraction of peptides that are exclusive to an allele in a specific sample, whereas other cells indicate the frequency of peptides shared among different samples and alleles. Peptides observed in control samples tend to be less frequently shared among different alleles and samples, while peptides from pulsed samples, despite their low frequencies, are distributed across a broader range of alleles and samples.

The recognition of the influence of dual peptides on allele behaviour within specific samples prompted us to conduct a separate overlap analysis of both peptides and PGPs across C‐DC and P‐DC samples, aiming to evaluate the impact of MoDC pulsing on peptide presentation. Heatmaps were generated to visualise the degree of overlap between alleles in terms of shared peptides (Figure 4) and shared PGPs (Figure S5), with higher values indicating greater overlap. For each allele, the intersection of both axes corresponds to the proportion of peptides or PGPs that are exclusive to that allele, that is, not shared with any other allele considered in the analysis.

In both sample groups, PGP overlap analysis revealed a reduced exclusivity for each allele (Figure S5). Notably, even no exclusive PGPs were identified for DRB1*03:01 in C‐2104, DRB1*07:01 in C‐2105, DRB1*03:01 in C‐2107 and DRB1*13:02 in P‐1914, suggesting a redundant protein presentation across alleles. Conversely, certain alleles exhibited increased peptide exclusivity, with fractions exceeding 0.5. These included DRB1*11:04 and DRB1*12:01 in C‐2103, DRB1*13:02 in C‐1921 and DRB1*07:01 in C‐1922 (Figure 4A), as well as DRB1*15:01 in P‐1902, DRB1*04:04 in P‐1903, DRB1*07:01 in P‐1907 and DRB1*01:02 in P‐1914 (Figure 4B). Interestingly, co‐expressed alleles within each sample frequently shared a substantial fraction of peptides, with overlap values exceeding 0.2 in most cases.

C‐DC's repertoires exhibited a substantial peptide overlap when samples shared at least one allele. For instance, DRB1*01:01 in C‐2102 mainly shared peptides with DRB1*01:01 and DRB1*07:01 in both C‐2105 and C‐2106, while in the homozygous C‐1922 sample, DRB1*07:01 exclusively shared peptides with DRB1*01:01 and DRB1*07:01 from other samples. This further supports the tendency of these alleles to present overlapping peptide repertoires. This effect was even more pronounced in heterozygous samples sharing the full HLA‐DR allele combination (C‐2105 and C‐2106), resulting in a highly overlapping peptide profile and reinforcing the influence of heterozygosity in these cases. A comparable pattern was observed in samples expressing DRB1*03:01 and DRB1*13 (C‐1921, C‐2104 and C‐2107), where peptide sharing occurred exclusively among these alleles, with no detectable overlap with others such as DRB1*01:01 and DRB1*07:01. In contrast, nearly all alleles in P‐DC samples exhibited peptide sharing with most other analysed alleles, consistent with the use of a common protein source for MoDC pulsing. This suggests that the addition of MCF‐7 extracts may have influenced peptide presentation by MoDCs.

### 3.5 Pulsing With MCF‐7 Extract Enhances Presentation of Cytosolic Proteins

**FIGURE 5:**
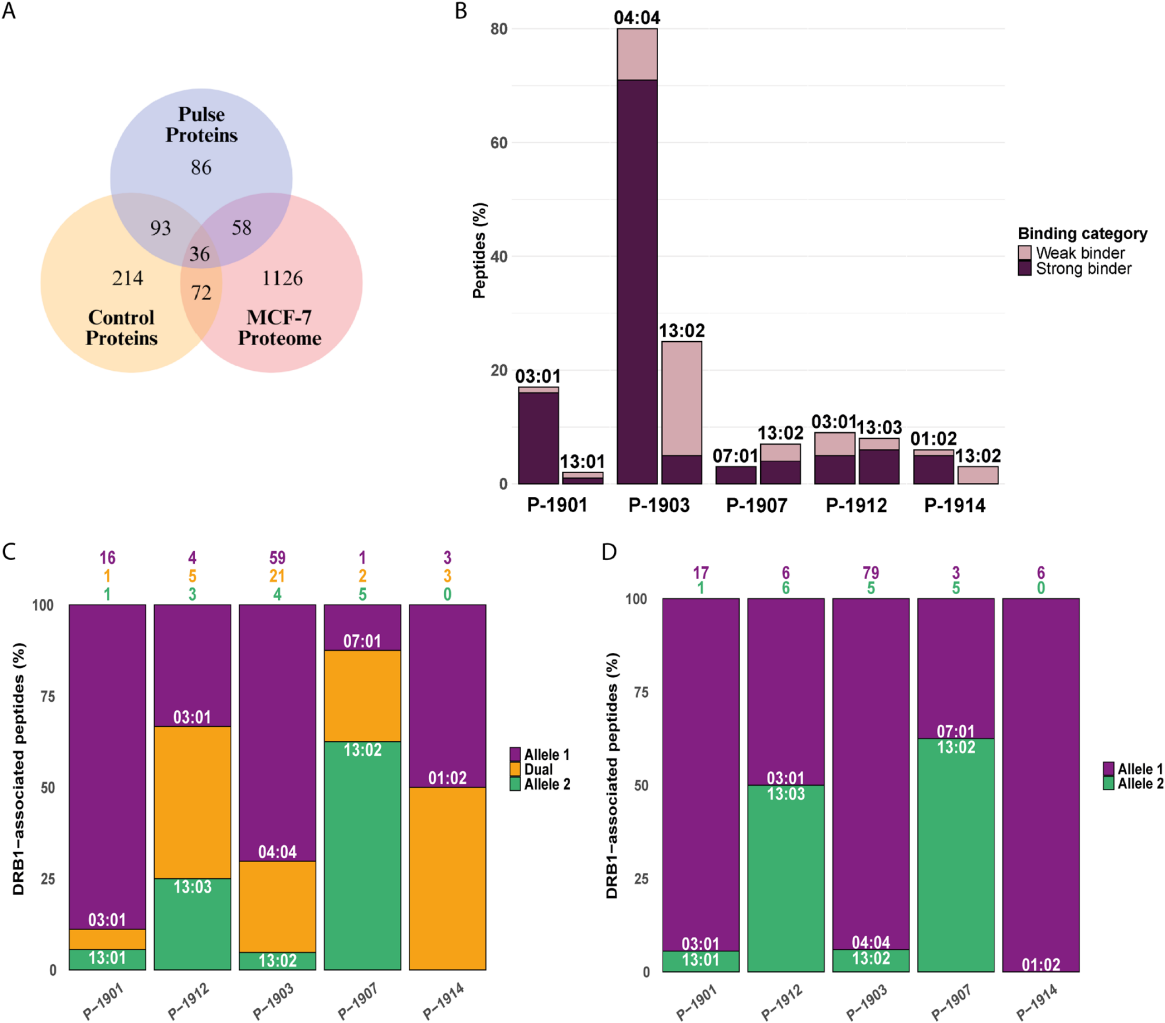
Peptide characterisation of MCF‐7‐induced peptides identified in P‐DC samples. (A) Venn diagram showing the overlap of proteins identified in pulsed (P‐) and control (C‐) DCs, as well as in the MCF‐7 proteome (the source of the lysate used for pulsing). Peptides derived from the 58 proteins shared between P‐DCs and the MCF‐7 proteome, but absent in C‐DCs, were classified as MCF‐7‐induced and used for subsequent analyses. (B) Peptide count of strong binders (SB) and weak binders (WB) associated with each allele in P‐DC samples, as determined by the affinity prediction algorithm of NetMHCIIPan4.1. While peptide identifications from the MCF‐7 extract were notably reduced in certain samples (e.g., P‐1907, P‐1912 and P‐1914), some alleles exhibit a marked increase in SB peptides (e.g., DRB1*0301 in P‐1901, DRB1*04:04 in P‐1903 and DRB1*01:02 in P‐1914), whereas others show a rise in WB peptides (e.g., DRB1*13:02 in both P‐1903 and P‐1914). (C) Percentage of MCF‐7‐induced peptides associated with each HLA‐DRB1 allele or both alleles (dual) in P‐DC samples. Some samples exhibit a strong bias towards a single allele (e.g., P‐1901 for *DRB103:01*, P‐1903 for DRB1*04:04, P‐1914 for DRB1*01:02), whereas others display a more balanced distribution (e.g., P‐1912, typed DRB1*03:01/*13:03). The proportion of dual‐assigned peptides varies significantly, ranging from over 50% in some cases (P‐1914) to less than 10% in others (P‐1901). (D) Same distribution as in (C) but with dual peptides assigned to the allele showing the highest predicted affinity score in each sample. The bias previously observed is further enhanced, resulting in over 90% of peptides being attributed to DRB1*03:01, DRB1*04:04 and DRB1*01:02 in samples P‐1901, P‐1902 and P‐1914, respectively.

The composition of the total MCF‐7 proteome is inherently shaped by the method used for lysate preparation (see Methods 3.1). We employed repeated freeze–thaw cycles and removed all nuclei and membranes by centrifugation to mimic a physiological pyroptotic and necrotic environment, in which cytosolic content is passively released, becoming accessible to DCs within the TME [37]. This approach aimed to simulate the type of antigenic material that DCs might encounter and uptake in vivo, thereby providing a relevant context for analysing antigen presentation under conditions resembling tumour‐associated necrosis. MS analysis of the MCF‐7 lysate identified 1292 different proteins (Table S3). Among these, 58 proteins (yielding 120 peptides) were uniquely detected in P‐DCs and absent in C‐DCs, suggesting that they were presumably derived from the MCF‐7 extract (Figure 5A) These 58 PGPs, hereafter referred to as ‘pulse‐induced proteins’ accounted for 4.5% of the entire MCF‐7 proteome and represented 21% of the immunopeptidome in the P‐DC sample set (see Tables S4 and S5 for details). Sample P‐1902, typed DRB1*13:05/*15:01, was excluded as no pulse‐induced proteins were identified. Interestingly, no positive correlation was observed between protein abundance in the MCF‐7 lysate and peptide representation in the P‐DC immunopeptidome, indicating that the most abundant proteins in the input material were not necessarily those most efficiently processed and presented by P‐DCs (Figure S6A). Furthermore, comparison of the subcellular localisation of pulse‐induced proteins to that of all PGPs identified in P‐DC samples revealed a significant enrichment in cytosolic proteins (*p* < 0.01), alongside a marked depletion of secreted and extracellular matrix‐derived proteins (*p* < 0.01) (Figure S6B). These shifts reinforce trends observed in the broader comparison between P‐DC and C‐DC protein repertoires (see Figure S3B), supporting the conclusion that MCF‐7 pulsing alters the immunopeptidome composition.

### 3.6 Pulse‐Driven Immunopeptidome Analysis Enhances Allele‐Specific Features and Reveals Distinct Binding Biases of the DRB1*03:01 and DRB1*13:02 Alleles in a Heterozygous Context

DRB1 affinity analysis of the 120 peptides derived from proteins present in the MCF‐7 lysate and detected after MoDC pulsing (hereafter referred to as MCF‐7‐induced peptides), revealed a pronounced increase in binding strength for DRB1*03:01 in P‐1901 and DRB1*04:04 in P‐1903 (Figure 5B), compared to peptides from the total protein pool in these samples (Figure S4). Furthermore, these alleles, along with DRB1*01:02 in P‐1914, demonstrated greater peptide exclusivity compared to their co‐expressed counterparts (Figure 5C). The dual peptide‐filtered distribution to one allele further underscores the dominant binding preference of DRB1*03:01, DRB1*04:04 and DRB1*01:02 over their DRB1*13 co‐expressed alleles, highlighting the peptide imbalance among alleles in the pulse‐induced proteins (Figure 5D). The extent of peptide sharing between DRB1*03:01 and its pairing alleles varied depending on the specific combination. In P‐1912 (DRB1*03:01/13:03), 55% (5 out of 9) of the MCF‐7‐induced peptides were shared between both alleles, whereas in P‐1901 (DRB1*03:01/13:01), 94.1% (16 out of 17) were exclusive to DRB1*03:01.

**FIGURE 6:**
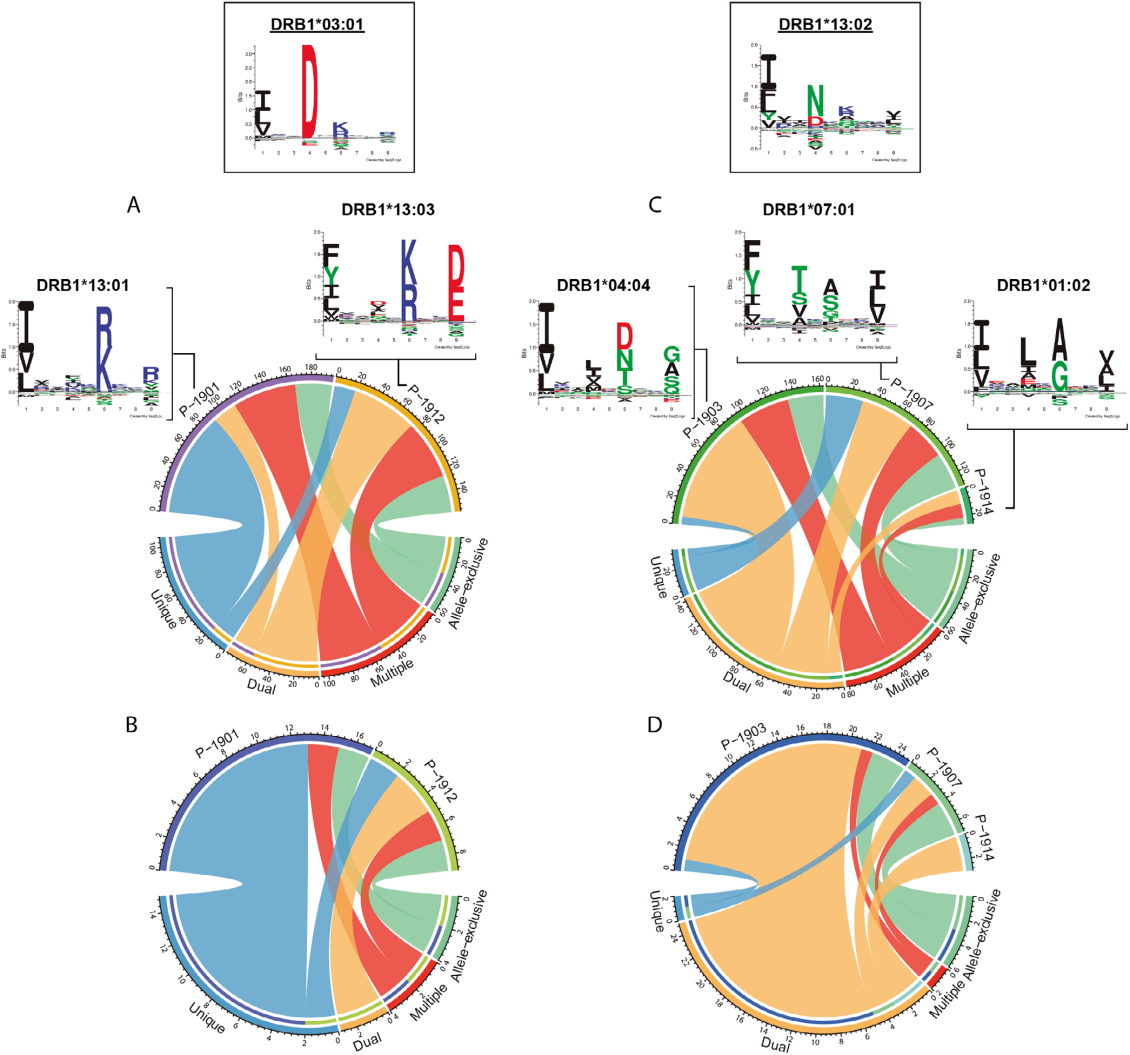
Distribution of peptides associated with HLA‐DRB1*03:01 and DRB1*13:02 in samples expressing these alleles, considering all peptides (A, C) or only MCF‐7‐induced peptides (B, D). The circular diagrams categorise peptides based on their occurrence: Peptides found exclusively in one sample and one allele (unique, blue), peptides present in both alleles within the same sample (dual, orange), peptides shared across multiple alleles in different samples (multiple, red) and peptides found in more than one sample but restricted to a single allele (allele‐exclusive, green). Distinct patterns are observed depending on the allelic combination. For instance, DRB1*03:01‐associated peptides in sample P‐1901 are predominantly unique. In sample P‐1912, a larger proportion of co‐expressed peptides is observed. In contrast, DRB1*13:02‐associated peptides are more frequently found in both alleles within a given sample in the three P‐DCs analysed. These differences likely stem from variations in the binding motif restrictions of these alleles, a trend that becomes more pronounced when considering only MCF‐7‐induced peptides. HLA‐specific core motifs are depicted at the figure top.

Next, the effect of heterozygosity was explored by focusing on the alleles common to more than one sample, specifically DRB1*03:01 and DRB1*13:02, restricting the analysis to the MCF‐7‐induced peptides and PGPs. For each allele, peptides were classified into four categories, based on their sharing status: (i) unique (exclusive to a given HLA‐DR allele in a single sample); (ii) dual (associated with the HLA‐DR allele and its paired allele within the same sample); (iii) multiple (associated with the HLA‐DR allele but also shared with other alleles across samples); and (iv) allele‐exclusive (associated with one HLA‐DR allele in more than one sample). Based on this classification, peptide distribution was separately analysed for all identified PGPs in P‐DC samples (Figure 6A,C) and for the 58 pulse‐induced proteins (Figure 6B,D). When considering all PGPs, DRB1*03:01 exhibited differential properties regarding the proportions of dual and unique peptides, depending on the allele combination. The proportion of dual peptides increased when combined with DRB1*13:03, as in P‐1912. In contrast, the number of unique peptides was notably higher in the case of DRB1*13:01 combination (Figure 6A). This analysis suggests a dominance effect of DRB1*03:01 over DRB1*13:01, as reflected in the MDS analysis, where the change in distance upon dual peptide filtering was evident (see Figure 3C). However, there was no dominance when DRB1*03:01 was co‐expressed with DRB1*13:03, likely due to the more similar binding core motives of these alleles. Both of their cores exhibit a selective preference for Asp in position P4, resulting in comparable peptide‐binding preferences and, consequently, in a higher proportion of dual peptides. Conversely, DRB1*13:02 exhibited a balanced peptide distribution when combined with DRB1*07:01 (P‐1907), while no unique peptide was identified for this allele in P‐1914, which presented fewer peptides overall. In contrast, about 50% of the peptides presented by DRB1*13:02 were shared with co‐expressed DRB1*04:04 in P‐1903 sample. In this specific combination, dual peptides exhibited stronger affinity for DRB1*04:04, resulting in the drag effect previously observed in the MDS analysis upon filtering dual peptides (Figure 6C).

Notably, the patterns observed in the previous analysis became more pronounced when only pulse‐induced proteins were considered (Figure 6B,D). This suggests that the pulse not only maintained but also accentuated the differential properties of the co‐expressed alleles. The enhanced effect was particularly evident in P‐1901 and P‐1903 samples, where only one allele (DRB1*03:01 and DRB1*04:04, respectively) exhibited increased dominance (Figure 5B,C).

### 3.7 HLA‐DRB1 Allelic Properties Impact the Presentation of MCF‐7‐Induced Peptides

Interestingly, DRB1 allelic differential properties were more manifest when analysing peptides derived from MCF‐7 proteins, including some involved in tumorigenesis. Annexin A2 (ANXA2), overexpressed in MCF‐7 cells [38], plays a role in tumour progression and in the response to immunotherapy [39, 40, 41]. The presentation of Annexin A2‐derived peptides was particularly evident in P‐1901, where three nested peptides sharing the IQQDTKGDY core were identified. All exhibited high predicted affinity for DRB1*03:01 but not for its co‐expressed allele, DRB1*13:01, highlighting the dominance of DRB1*03:01 in this sample. Another key protein, Galectin‐1 (LGALS1), which is also relevant to breast cancer and to MCF‐7 cell proliferation [42, 43], and more recently implicated in pancreatic ductal adenocarcinoma progression [44], generated a single peptide exclusively presented by DRB1*03:01 in both P‐1901 and P‐1912.

In turn, DRB1*13:02 exhibited a reduced immunopeptidome when co‐expressed with DRB1*01:02 and DRB1*04:04, but not with DRB1*07:01. In P‐1914, no exclusive peptides were detected for DRB1*13:02. In P‐1903, only 16% (4 out of 25) of peptides attributed to DRB1*13:02 were both exclusive and SB, all belonging to a single nested set with the core sequence IILNHPGQI, derived from elongation factor 1‐alpha 2 (EEF1A2). In P‐1907, although few MCF‐7‐induced peptides were identified overall, the same nested set from EEF1A2 contributed to the exclusivity of DRB1*13:02 in this sample. Despite the generally weak binding tendency of DRB1*13:02 observed in P‐1914 and P‐1903, it was still capable of selectively presenting certain relevant proteins such as EEF1A2. This protein is considered proto‐oncogenic [45, 46] and is overexpressed in around 60% primary breast tumours, particularly in oestrogen receptor‐positive cases [47]. However, its prognostic significance remains complex and potentially dependent on the specific breast cancer subtype [48, 49].

## 4 Discussion

CD4+ T cells are key orchestrators of anti‐tumour immunity, enhancing the activation of cytotoxic T cells and promoting immunological memory [50]. The presentation of tumour‐derived antigens by MHC class II molecules shapes the TCR repertoire of intratumor CD4+ T cells, directly influencing their role in tumour immunity. However, the role of the MHC‐II immunopeptidome in heterozygous individuals remains poorly understood, which limits our understanding of how allele co‐expression modulates peptide presentation.

Within class II immunopeptidomes, HLA‐DR‐associated peptides have been the most extensively characterised. The HLA‐DR repertoire is shaped by molecules using beta chains encoded by the DRB1, DRB3, DRB4 and DRB5 genes. DRB1 alleles account for a large proportion of the detected HLA‐DR peptidome as they are always present and therefore, in the present study, we did not extend the analysis to explicitly dissect the contribution of secondary HLA‐DR haplotypes (HLA‐DR51, ‐DR52, ‐DR53). Moreover, the limited number of donors carrying these haplotypes in our samples precluded robust conclusions regarding these alleles. In addition, the strong linkage disequilibrium of these secondary loci with DRB1, together with their lower degree of polymorphism, constrains the evaluation of heterozygosity for these genes [51]. However, it should be noted that secondary DRB loci could also substantially shape antigen presentation in specific haplotypes, that is, DRB5 can strongly influence the HLA‐DR peptide repertoire in DR51 haplotypes, DRB4 generally contributes in more limited manner in DR53 haplotypes, and DRB3 shows variable contributions in DR52 haplotypes, depending on the associated DRB1 allele [20].

Considering that the monoclonal antibody used for immunoprecipitations (clone L243) captures all HLA‐DR molecules and therefore elutes peptides bound to both primary and secondary DRB products, to avoid misassignment and overinterpretation, downstream analyses were therefore intentionally restricted to peptides predicted to bind donor‐matched HLA‐DRB1 alleles. This approach allowed us to robustly interrogate DRB1‐associated presentation patterns within the physiologically relevant competitive class II environment present in each sample, while acknowledging that peptides presented by secondary DRB molecules were not individually resolved. We also did not include HLA‐DP and HLA‐DQ alleles in this initial study, as this was the first time we examined the behaviour of allelic pairs within a single type of molecule. Given the complexity of this analysis, we restricted our focus to HLA‐DR, which is also the most extensively characterised HLA class II molecule. Nevertheless, extending this approach to HLA‐DP and HLA‐DQ would be of interest in future studies.

To assess the impact of heterozygosity on peptide availability for presentation across a set of HLA‐DRB1 alleles, we analysed the MHC‐II immunopeptidome of 13 heterozygous C‐DC and P‐DC samples, expressing 13 distinct DRB1 alleles that formed 11 unique combinations. DRB1*03:01/*13:03 and DRB1*01:01/*07:01 combinations were each expressed by two samples. HLA typing was performed retrospectively, after donor selection, which had been carried out blindly with respect to HLA type to avoid introducing selection bias into the study. The presence of DRB1*01:01, DRB1*03:01 and DRB1*13:02 alleles in several samples enabled us to identify differential properties associated with the co‐expression of other alleles.

The presence of nested‐set peptides in class II immunopeptidomes is a classical feature of class II peptides that reduces the diversity of presented proteins. This was thoroughly studied by Mommen et al., who provided an in‐depth analysis of the HLA class II ligandome of DCs, revealing that the 100 most prevalent proteins accounted for 80% of the peptides presented by the HLA‐DR molecules in a cell, with 84% of the peptides distributed into nested‐sets [52]. This, combined with the higher number of anchor residues required for MHC‐II binding (mainly P1, P4, P6 and P9 in MHC‐II vs. P2 and P9 in MHC‐I) and the effect of PFRs, further constrains the MHC‐II ligandome, making it more limited than that of MHC‐I. Our data confirm the high prevalence of nested‐set peptides, suggesting that class II molecules may become saturated, thereby conditioning the antigen dose required for effective CD4+ T cell activation.

During peptide assignment to HLA‐DRB1 alleles, a specific imbalance between the two alleles within each sample emerged. This disparity was particularly prominent in P‐1901, P‐1903 and C‐2102 samples, where alleles DRB1*03:01, DRB1*04:04 and DRB1*01:01, respectively, were predicted to bind approximately three times more peptides than their co‐expressed alleles (DRB1*13:01, DRB1*13:02 and DRB1*11:01, respectively). While only a limited number of MS‐based immunopeptidomics studies have directly investigated allele‐specific differences in peptide presentation, some of these characteristics are consistently reflected in a parallel study using the same antibody [53], which analysed the natural ligandome of five distinct thymus DRB1*03:01‐positive samples.

The observed differences in peptide assignment are unlikely to result from the use of L243 anti‐HLA‐DR antibody, which recognises a conformational epitope on the HLA‐DR α chain and does not exhibit DRB1‐specific bias recovery. Instead, differential expression levels of HLA‐DRB1 alleles may contribute to this imbalance. In healthy individuals, DRB1*04:04 and *07 alleles are associated with higher DRB1 expression compared to DRB1*15 alleles, a trend also observed at the protein level [54]. This variation could partially explain the higher peptide recovery in sample P‐1903 (expressing DRB1*04:04) compared to P‐1902 (expressing DRB1*15:01). Beyond expression levels, variations in peptide representation may also be influenced by differences in CLIP binding affinity and HLA‐DM dependence among HLA‐DR alleles [55, 56]. HLA‐DM acts as a sensor of MHC–peptide complex stability, playing a crucial role in replacing CLIP with high‐affinity peptides [57]; however, recent findings suggest that polymorphisms within the peptide‐binding groove of HLA‐DR alleles can induce allosteric conformational changes at the HLA‐DM interaction site (α‐DM interface), thereby modulating the efficiency of peptide exchange [17]. Notably, DRB1*13:02 has been reported to exhibit significantly high CLIP affinity [55], making it more resistant to peptide loading and potentially affecting CD4+ T cell responses. Conversely, DRB1*04:04 forms a CLIP complex with low thermal stability and high susceptibility to HLA‐DM‐mediated peptide exchange [17], which may contribute to its increased peptide recovery in P‐1903 sample. In cancer contexts, CLIP removal may be impaired, leading to defective class II antigen presentation and ultimately hindering CD4+ T cell activation, thereby compromising anti‐tumour immune responses, as described in an experimental mouse model [22]. HLA‐DO can further modulate peptide loading by interacting with HLA‐DM through a substrate‐mimicry mechanism that competitively limits HLA‐DM's catalytic activity [58], thereby stabilising CLIP‐bound complexes and reducing peptide exchange efficiency. Several studies have shown that HLA‐DO expression gets downregulated during B cell development [59] and during DC maturation [60], which in turn favours the loading of immunodominant, high‐stability peptides onto MHC‐II molecules. Although transcriptomic analyses reveal that class II gene expression can vary across individuals [54], no consistent allele‐specific differences in HLA‐DM or HLA‐DO expression levels linked to DRB have been reported to date.

Recent large‐scale immunopeptidomics studies [29, 61] have classified HLA‐II alleles into isotypes based on similar peptide‐binding motifs, suggesting that certain allele combinations may share a greater proportion of presented peptides or even compete for them. However, experimental validation is still needed to fully understand how different allele co‐expression shapes the HLA‐II repertoire. In this study, we demonstrate that heterozygosity affects peptide presentation in an allele‐specific manner, with consistent allele behaviour across repeated combinations (*n* = 2). DRB1*01:01/*07:01 pairs exhibited similar presentation patterns due to their similar and less restrictive binding motifs, as similarly evidenced by Ciudad et al. [36]. DRB1*03:01/*13:03 demonstrated a selective preference for Asp at P4, yielding an overlapping peptide repertoire. However, the behaviour of alleles belonging to the same isotype varied depending on the co‐expressed alleles. Immunopeptidomics analysis of thymus tissue from Collado et al. [53] co‐expressing DRB1*01:01/*03:01 (*n* = 2) and DRB1*03:01/*11:01 (*n* = 2), revealed a certain dominance of DRB1*01:01 and DRB1*11:01 over DRB1*03:01. In these cases, more than 50% of the peptides were assigned to the dominant allele, whereas fewer than 15% were associated with DRB1*03:01. Despite the lower DRB1*03:01 contribution in these combinations, it behaves as dominant when co‐expressed with DRB1*15:01 [53, 62]. In our data, DRB1*03:01 was dominant with DRB1*13:01 but not with DRB1*13:03, while DRB1*01:01 dominated in combination with DRB1*11:01, but not with DRB1*07:01. Notably, DRB1*13:02 consistently exhibited a weak binding capacity, except when co‐expressed with DRB1*13:01.

These differences in allelic dominance directly influenced the presence of dual peptides, which may arise from degenerate peptides containing binding cores capable of interacting with multiple alleles [63, 64]. The longer length of class II peptides, compared to class I ligands, further increases the likelihood of identifying distinct binding cores within a single peptide compatible with different alleles, as previously demonstrated for the dominant CD4+ T cell epitope from SSX‐2 antigen [65]. In our study, dual peptides served as a valuable tool for assessing allelic interactions in various combinations. To ensure accurate peptide assignment, we used NetMHCIIPan4.1, as other affinity prediction algorithms, such as MHCMotifDecon‐1.0 [20], restrict peptide assignment to a single allele, disregarding the possibility that peptides could bind concomitantly to the two co‐expressed alleles. MDS analysis, filtering out or not dual peptides, demonstrated that peptide presentation is influenced by both intrinsic allele properties and specific allelic pairing.

The limited MoDC recovery after peptide pulsing prevented the use of the same sample for both pulse and non‐pulse conditions; however, using separate samples increased biological variability to the study design. When analysing the effect of pulsing P‐DCs, we observed a moderate increase in peptide overlap between alleles, which contrasts with C‐DC samples, where only certain alleles shared peptides. However, the highest overlap was found between co‐expressed alleles within individual samples (reinforcing the concept of dual peptides) and between samples with identical allele combinations (C‐2105 and C‐2106). PGPs overlap was even greater, suggesting comparable protein processing and presentation, aligning with the previously discussed hypothesis by Mommen et al. [52]. Among the entire proteome derived from the MCF‐7 tumour cell line, only 4.5% was exclusively presented by P‐DC samples after discarding peptides found in C‐DC samples. Despite the limited number of pulse‐induced proteins identified, both allele‐specific characteristics and co‐expression effects were preserved or even accentuated, particularly in DRB1*03:01 (dominance) and DRB1*13:02 (weakness) alleles when combined with DRB1*13:01 in P‐1901 and DRB1*04:04 in P‐1903, respectively. This analysis further illustrates how the behaviour of individual DRB1 alleles, modulated by their allelic context, directs antigen presentation.

Immunodominance has been well established for HLA class I alleles, which present highly immunogenic viral epitopes, shaping CD8+ T cell responses. In the context of anti‐tumour immune responses, several tumour‐associated antigens have been described as potential immunogenic molecules. Specifically, HLA‐A*02:01 has been reported to present epitopes from MAGE [66], NY‐ESO [67], WT‐1 [68], GP100 [69] and MART1 [70]. In contrast, evidence for HLA class II immunodominance, particularly in the tumour context, remains limited [71, 72], with several described epitopes corresponding to class I‐derived cores flanked by additional residues [73]. This gap highlights the need to better understand how HLA class II molecules shape antigen presentation in tumours and their potential involvement in CD4+ T cell immunodominance.

Finally, MCF‐7‐induced peptides may represent either direct presentation of tumour‐associated proteins or indirect effects of tumour lysate exposure on antigen processing. In our peptide analysis, we could not assign immunodominant properties to specific peptides. However, by identifying MCF‐7‐induced peptides from proteins such as Annexin A2, Galectin‐1 and EEF1A2, known to be relevant in breast cancer, we demonstrated that allele‐specific behaviour is also observed for tumour antigens. The presentation of EEF1A2 by DRB1*13:02 in two different samples, despite co‐expression with immunodominant alleles, suggests that in DRB1*13:02‐positive individuals with tumours overexpressing this protein, EEF1A2‐derived peptides could represent a potential therapeutic target. Moreover, high expression levels of EEF1A2 have been associated with an enrichment of tumour‐associated CD4^+^ memory T cells [74], further supporting its immunological relevance in the TME.

In the present work, we have performed a comprehensive analysis of immunopeptidomics data collected from six MCF‐7 pulsed and eight non‐pulsed MoDC samples, revealing distinct allele‐specific behaviour and a significant influence of heterozygosity on peptide presentation patterns. The analysis demonstrates that alleles with high binding strength tend to play a dominant role in antigen presentation in heterozygous individuals, making them advantageous for immunotherapy strategies aimed at broadening peptide presentation and immune recognition. Conversely, alleles with lower binding strength could contribute to a more selective immune response. In summary, our findings provide new insights into antigen presentation patterns in heterozygous individuals, pointing to one additional variable in designing personalised immunotherapies.

## Author Contributions

Conceptualisation: A.A., M.M. Methodology: G.L., J.A.C., M.F. and A.A. Investigation: G.L., J.A.C., M.F., C.R.‐M., L.G., J.P.‐G., J.R.‐G., J.C., A.A. and M.M. Resources: C.R.‐M. Writing – original draft: G.L., A.A. and M.M. Writing – review and editing: G.L., A.A. and M.M. Supervision: A.A. and M.M. All authors contributed to the manuscript and approved the submitted version.

## Funding

This project was funded by the Contigo Contra el Cáncer de la Mujer Foundation (#BREASTILs Project), ‘Functional Study of TILs from breast cancer patients: an approach to personalized medicine’, by the Fundación de Investigación Oncológica (FERO) and the Spanish Ministry of Science, Innovation and Universities grant PID2021‐125470OB‐I00. Spanish Ministry of Science, Innovation and Universities was not involved in the study design, collection, analysis, interpretation of data, the writing of this article, or the decision to submit it for publication.

## Disclosure

All claims expressed in this article are solely those of the authors and do not necessarily represent those of their affiliated organisations, or those of the publisher, the editors and the reviewers. Any product that may be evaluated in this article, or claim that may be made by its manufacturer, is not guaranteed or endorsed by the publisher.

## Ethics Statement

Leukocyte residues from 14 healthy donors were obtained from the Biobanco del Centro de Hemoterapia y Hemodonación (Valladolid, Spain), with the appropriate approval of the Ethical and Scientific Committee of the institution. Consent was obtained from donors according to the local institutional review board requirements.

## Conflicts of Interest

The authors declare no conflicts of interest.

## Supporting information


**Figure S1:** Computational analysis framework for studying the role of HLA‐DR heterozygosity in antigen presentation. (1) The pipeline begins with the collection of peptide sequences presented by individuals with known HLA types. (2) A distance matrix is computed based on global sequence alignments using BLOSUM62 matrix to evaluate peptide similarity. (3) The optimal number of clusters is determined to group peptides that are homologous or belong to nested sets, reducing complexity. These peptide clusters are then associated with HLA alleles. Peptides capable of binding to both co‐expressed alleles, that is, dual peptides, were identified in all samples. (4) The analysis proceeds in two parallel paths: in the filtered approach, each dual peptide in heterozygous individuals is assigned to the allele with the highest predicted binding affinity; in the non‐filtered approach, dual peptides are assumed to bind equally to both co‐expressed alleles. Multidimensional scaling (MDS) is applied to both datasets to visualise the spatial relationships among HLA‐DRB1 alleles based on the peptide clusters they present. (5) The distributions from the filtered and non‐filtered approaches are compared to evaluate how different assignment strategies affect the interpretation of allele‐specific peptide presentation. MDS, multidimensional scaling.
**Figure S2:** Phenotypic characterisation of mature MoDCs prior to HLA‐DR‐peptide immunoprecipitation. (A) Representative flow cytometry histograms from a single MoDC sample showing high fluorescence intensity for HLA‐DR and additional DC maturation markers. The gating strategy was based on a negative control, resulting in nearly 100% positivity for all markers in the tested samples. (B) Bar plots representing the percentage of positive cells for each marker (HLA‐ABC, HLA‐DR, CD80, CD83 and CD86), shown as mean ± standard deviation, separately for both control and pulsed samples. No statistical significance was observed between conditions, suggesting that pulsing did not influence the maturation status of MoDCs. Nearly all MoDCs express surface HLA‐DR, ensuring the presence of DR‐bound peptides, along with high expression of other activation markers.
**Figure S3:** General characterisation of HLA‐DR eluted peptides in control (C‐DC) and pulsed (P‐DC) samples. (A) Peptide length showing a normal distribution with an average of 15‐mers in both groups of samples. (B) Subcellular distribution of the source proteins. Data are presented as mean ± standard deviation (SD) for each subcellular compartment in both conditions. Multiple unpaired *t*‐tests were performed for comparisons between conditions without assuming equal variances. Statistical significance was determined using the Holm‐Sidak method to correct for multiple comparisons, with a significance level of α = 0.05. Each compartment was analysed independently. A moderate increase in the percentage of nuclear and mitochondrial proteins was observed in pulsed samples, along with a decrease in proteins from the cell membrane and extracellular compartments. CM, cell membrane; S/EM, secreted or extracellular matrix; C, cytosol; N, nucleus; Lys/End, lysosome or endosome; ER, endoplasmic reticulum; GA, Golgi apparatus; Mit, mitochondria. *p < 0.05, **p < 0.01.
**Figure S4:** Percentage of strong binders (SB) and weak binders (WB) associated with each HLA‐DRB1 allele in P‐DC (A) and C‐DC (B) samples, determined by the affinity prediction algorithm of NetMHCIIPan4.1. Certain alleles (e.g., DRB1*01:01, DRB1*01:02 and DRB1*04:04 in C‐2102, P‐1914 and P‐1903 respectively) displayed a stronger affinity for binding peptides compared to others (e.g., DRB1*11:01, DRB1*13:01, DRB1*13:02 and DRB1*13:05 in C‐2102, P‐1901/C‐1921, P‐1903 and P‐1902, respectively).
**Figure S5:** Heatmap showing the frequency of protein overlap between DC control (A) and pulsed (B) samples and alleles. Diagonal cells display the fraction of proteins that are exclusive to an allele in a specific sample, whereas other cells indicate the frequency of proteins shared among different samples and alleles. Proteins observed in both control and pulsed samples tend to be frequently shared among different alleles and samples, with a lower frequency of protein exclusivity in most cases.
**Figure S6:** Protein characterisation of MCF‐7 protein lysates. (A) Relationship between the most abundant proteins in the MCF‐7 proteome and their peptide representation in the P‐DC immunopeptidomes. No positive correlation was observed, indicating that highly prevalent proteins in the MCF‐7 proteome were not necessarily the most represented in the P‐DC samples, and vice versa, and suggesting that protein abundance alone does not dictate peptide presentation in P‐DCs. Complete names of the proteins are collected in Table S5. (B) Subcellular distribution of pulse‐derived proteins compared to the overall protein composition in P‐DC samples. Data are presented as mean ± standard deviation (SD) for each subcellular compartment in both conditions. Multiple unpaired *t*‐tests were performed for comparisons between conditions without assuming equal variances. Statistical significance was determined using the Holm‐Sidak method to correct for multiple comparisons, with a significance level of α = 0.05. Each compartment was analysed independently. Pulse‐derived proteins showed a significant increase in the proportion of cytosolic proteins, along with contributions from other cellular compartments (e.g., GA, Mit), while there was a notable decrease in proteins from the secreted or extracellular matrix compartments. CM, cell membrane; S/EM, secreted or extracellular matrix; C, cytosol; N, nucleus; Lys/End, lysosome or endosome; ER, endoplasmic reticulum; GA, Golgi apparatus; Mit, mitochondria. *p < 0.05, **p < 0.01.

**Table S1:** Summary table showing the number of total, filtered, binder, and dual peptides identified in each sample, along with the number of peptides associated with each HLA‐DRB1 allele. The number of MoDCs obtained per sample ranged from 1.55 to 17.3 million. Peptide binding affinities for the different alleles were predicted using the NetMHCIIPan4.1 algorithm, revealing allele‐specific binding imbalances.

**Table S2:** (A) List of all peptides eluted from HLA‐DR molecules in MoDC samples. (B) List of peptides assigned to HLA‐DRB1 alleles in each MoDC sample. (C) List of all source proteins identified in MoDC samples. (D) List of source proteins assigned to HLA‐DRB1 alleles.

**Table S3:** List of proteins identified in the MCF‐7 lysates used for MoDC pulsing.

**Table S4:** Summary table showing the number of pulse‐derived peptides and proteins associated with each HLA‐DRB1 allele in every sample. P‐DC1902 was excluded as no pulse‐derived proteins were identified.

**Table S5:** (A) List of pulse‐derived peptides assigned to HLA‐DRB1 alleles in each P‐DC sample. (B) List of pulse‐derived proteins assigned to HLA‐DRB1 alleles in each P‐DC sample.

## Data Availability

The datasets presented in this study can be found in online repositories. The mass spectrometry proteomics data have been deposited to the ProteomeXchange Consortium via the PRIDE partner repository [75] with the dataset identifiers PXD064951 and PXD064999.
