# Supplementary material for: HLA‐DRB1 Allelic Combinations Differentially Shape Dendritic Cell Antigen Presentation Enhanced by Tumour Cell Line Lysate‐Pulsing
Source: HLA. 2026 Jan 28;107(2):e70563. doi: 10.1111/tan.70563 (PMC12853012; doi:10.1111/tan.70563)
Supplement: Supplementary file 1 — Figure S1: Computational analysis framework for studying the role of HLA‐DR heterozygosity in antigen presentation. (1) The pipeline begins with the collection of peptide sequences presented by individuals with known HLA types. (2) A distance matrix is computed based on global sequence alignments using BLOSUM62 matrix to evaluate peptide similarity. (3) The optimal number of clusters is determined to group peptides that are homologous or belong to nested sets, reducing complexity. These peptide clusters are then associated with HLA alleles. Peptides capable of binding to both co‐expressed alleles, that is, dual peptides, were identified in all samples. (4) The analysis proceeds in two parallel paths: in the filtered approach, each dual peptide in heterozygous individuals is assigned to the allele with the highest predicted binding affinity; in the non‐filtered approach, dual peptides are assumed to bind equally to both co‐expressed alleles. Multidimensional scaling (MDS) is applied to both datasets to visualise the spatial relationships among HLA‐DRB1 alleles based on the peptide clusters they present. (5) The distributions from the filtered and non‐filtered approaches are compared to evaluate how different assignment strategies affect the interpretation of allele‐specific peptide presentation. MDS, multidimensional scaling. Figure S2: Phenotypic characterisation of mature MoDCs prior to HLA‐DR‐peptide immunoprecipitation. (A) Representative flow cytometry histograms from a single MoDC sample showing high fluorescence intensity for HLA‐DR and additional DC maturation markers. The gating strategy was based on a negative control, resulting in nearly 100% positivity for all markers in the tested samples. (B) Bar plots representing the percentage of positive cells for each marker (HLA‐ABC, HLA‐DR, CD80, CD83 and CD86), shown as mean ± standard deviation, separately for both control and pulsed samples. No statistical significance was observed between conditions, suggesti [file TAN-107-e70563-s003.docx]

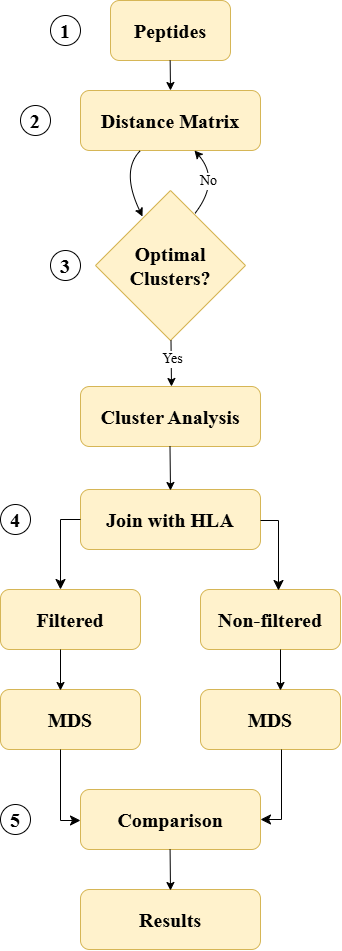


**Supplementary Figure 1. Computational analysis framework for studying the role of HLA-DR heterozygosity in antigen presentation.** (1) The pipeline begins with the collection of peptide sequences presented by individuals with known HLA types. (2) A distance matrix is computed based on global sequence alignments using BLOSUM62 matrix to evaluate peptide similarity. (3) The optimal number of clusters is determined to group peptides that are homologous or belong to nested sets, reducing complexity. These peptide clusters are then associated with HLA alleles. Peptides capable of binding to both co-expressed alleles, i.e., dual peptides, were identified in all samples. (4) The analysis proceeds in two parallel paths: in the filtered approach, each dual peptide in heterozygous individuals is assigned to the allele with the highest predicted binding affinity; in the non-filtered approach, dual peptides are assumed to bind equally to both co-expressed alleles. Multidimensional scaling (MDS) is applied to both datasets to visualize the spatial relationships among HLA-DRB1 alleles based on the peptide clusters they present. (5) The distributions from the filtered and non-filtered approaches are compared to evaluate how different assignment strategies affect the interpretation of allele-specific peptide presentation. MDS, multidimensional scaling.


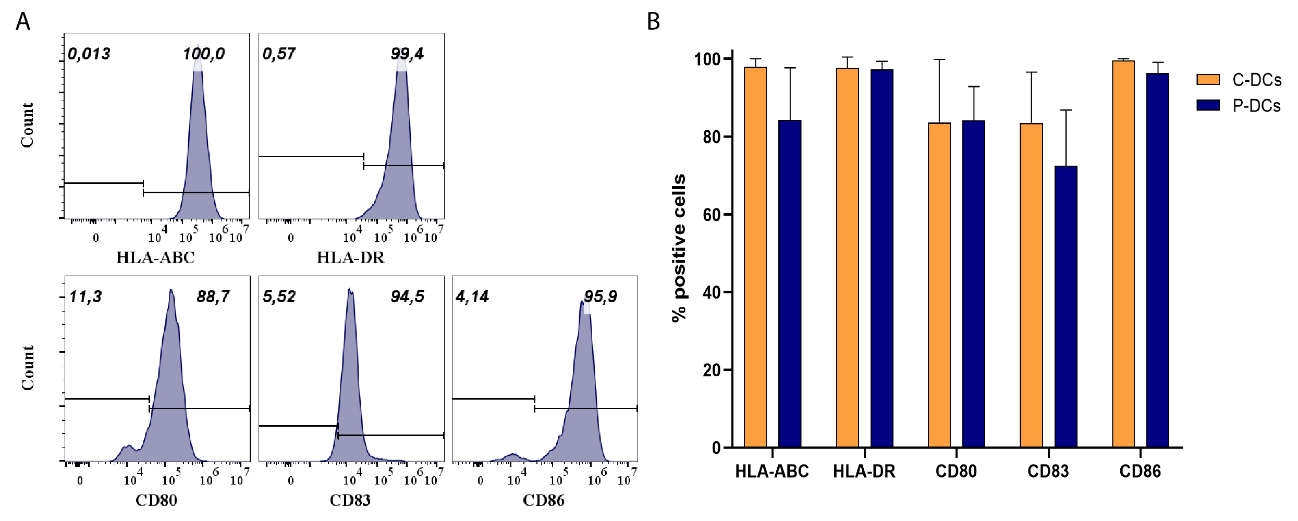


**Supplementary Figure 2. Phenotypic characterization of mature MoDCs prior to HLA-DR-peptide immunoprecipitation.** (A) Representative flow cytometry histograms from a single MoDC sample showing high fluorescence intensity for HLA-DR and additional DC maturation markers. The gating strategy was based on a negative control, resulting in nearly 100% positivity for all markers in the tested samples. (B) Bar plots representing the percentage of positive cells for each marker (HLA-ABC, HLA-DR, CD80, CD83 and CD86), shown as mean ± standard deviation, separately for both control and pulsed samples. No statistical significance was observed between conditions, suggesting that pulsing did not influence the maturation status of MoDCs. Nearly all MoDCs express surface HLA-DR, ensuring the presence of DR-bound peptides, along with high expression of other activation markers.


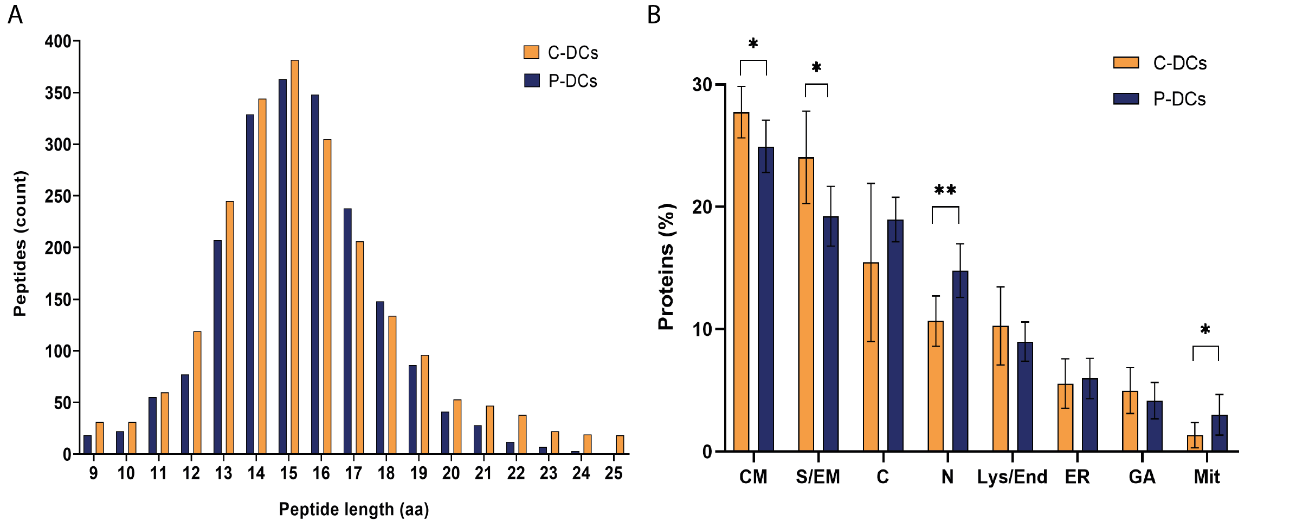


**Supplementary Figure 3. General characterization of HLA-DR eluted peptides in control (C-DC) and pulsed (P-DC) samples.** (A) Peptide length showing a normal distribution with an average of 15-mers in both groups of samples. (B) Subcellular distribution of the source proteins. Data are presented as mean ± standard deviation (SD) for each subcellular compartment in both conditions. Multiple unpaired t-tests were performed for comparisons between conditions without assuming equal variances. Statistical significance was determined using the Holm-Sidak method to correct for multiple comparisons, with a significance level of α = 0.05. Each compartment was analyzed independently. A moderate increase in the percentage of nuclear and mitochondrial proteins was observed in pulsed samples, along with a decrease in proteins from the cell membrane and extracellular compartments. CM, cell membrane; S/EM, secreted or extracellular matrix; C, cytosol; N, nucleus; Lys/End, lysosome or endosome; ER, endoplasmic reticulum; GA, Golgi apparatus; Mit, mitochondria. *p < 0.05, **p < 0.01.


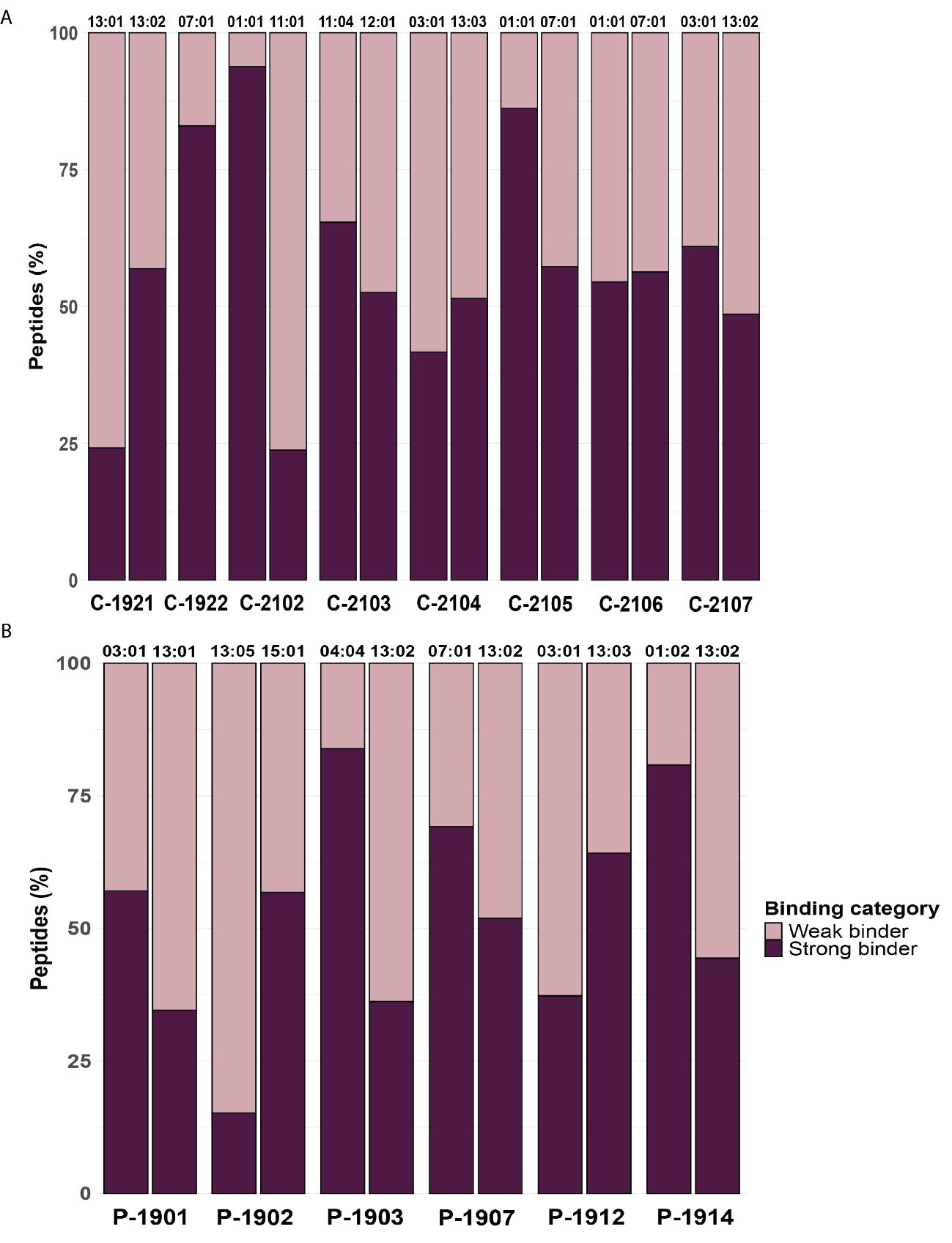


**Supplementary Figure 4. Percentage of strong binders (SB) and weak binders (WB) associated with each HLA-DRB1 allele in P-DC (A) and C-DC (B) samples, determined by the affinity prediction algorithm of NetMHCIIPan4.1**. Certain alleles (e.g., DRB1*01:01, DRB1*01:02 and DRB1*04:04 in C-2102, P-1914 and P-1903 respectively) displayed a stronger affinity for binding peptides compared to others (e.g., DRB1*11:01, DRB1*13:01, DRB1*13:02 and DRB1*13:05 in C-2102, P-1901/C-1921, P-1903 and P-1902, respectively).


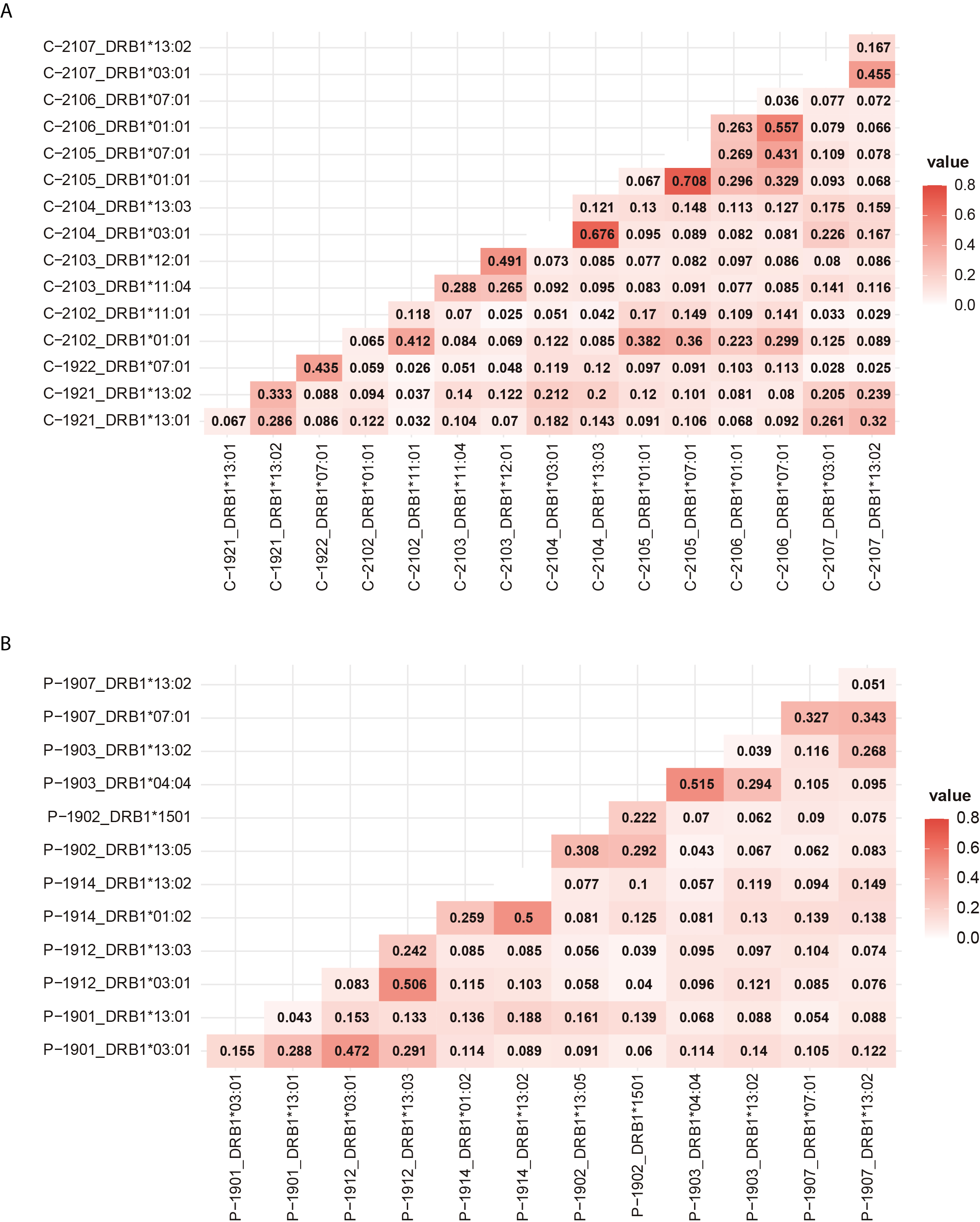


**Supplementary Figure 5. Heatmap showing the frequency of protein overlap between DC control (A) and pulsed (B) samples and alleles.** Diagonal cells display the fraction of proteins that are exclusive to an allele in a specific sample, whereas other cells indicate the frequency of proteins shared among different samples and alleles. Proteins observed in both control and pulsed samples tend to be frequently shared among different alleles and samples, with a lower frequency of protein exclusivity in most cases.


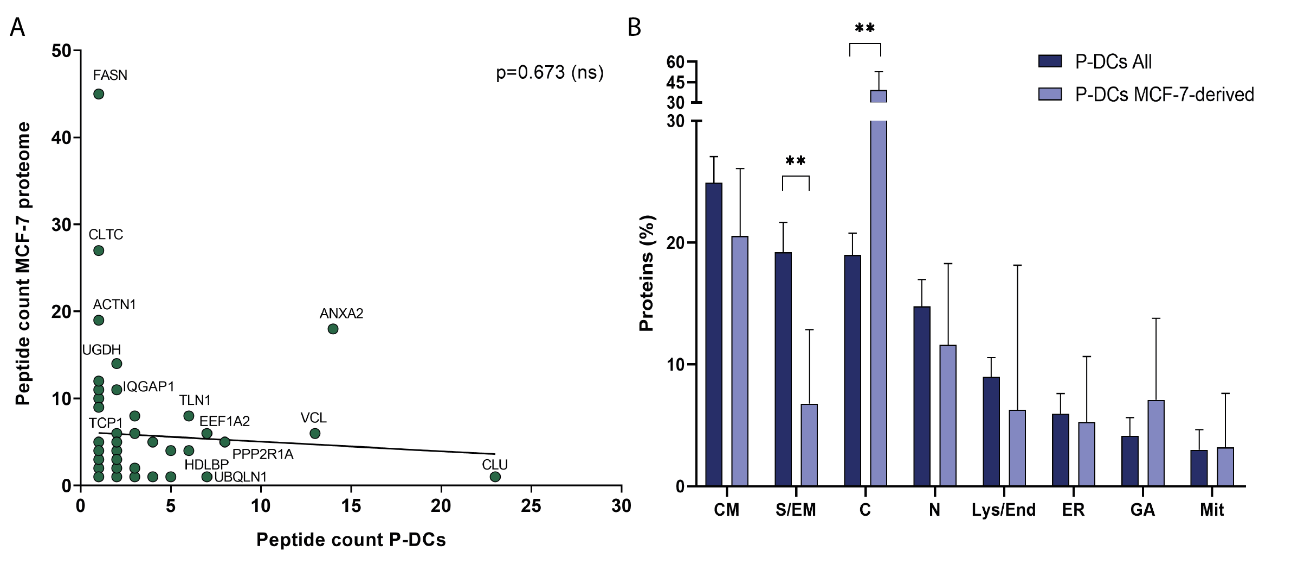


**Supplementary Figure 6. Protein characterization of MCF-7 protein lysates.** (A) Relationship between the most abundant proteins in the MCF-7 proteome and their peptide representation in the P-DC immunopeptidomes. No positive correlation was observed, indicating that highly prevalent proteins in the MCF-7 proteome were not necessarily the most represented in the P-DC samples, and vice versa, and suggesting that protein abundance alone does not dictate peptide presentation in P-DCs. Complete names of the proteins are collected in Supplementary Table 5. (B) Subcellular distribution of pulse-derived proteins compared to the overall protein composition in P-DC samples. Data are presented as mean ± standard deviation (SD) for each subcellular compartment in both conditions. Multiple unpaired t-tests were performed for comparisons between conditions without assuming equal variances. Statistical significance was determined using the Holm-Sidak method to correct for multiple comparisons, with a significance level of α = 0.05. Each compartment was analyzed independently. Pulse-derived proteins showed a significant increase in the proportion of cytosolic proteins, along with contributions from other cellular compartments (e.g., GA, Mit), while there was a notable decrease in proteins from the secreted or extracellular matrix compartments. CM, cell membrane; S/EM, secreted or extracellular matrix; C, cytosol; N, nucleus; Lys/End, lysosome or endosome; ER, endoplasmic reticulum; GA, Golgi apparatus; Mit, mitochondria. *p < 0.05, **p < 0.01.
