# Supplementary material for: HLA‐DRB1 Allelic Combinations Differentially Shape Dendritic Cell Antigen Presentation Enhanced by Tumour Cell Line Lysate‐Pulsing
Source: HLA. 2026 Jan 28;107(2):e70563. doi: 10.1111/tan.70563 (PMC12853012; doi:10.1111/tan.70563)
Supplement: Supplementary file 2 — Table S1: Summary table showing the number of total, filtered, binder, and dual peptides identified in each sample, along with the number of peptides associated with each HLA‐DRB1 allele. The number of MoDCs obtained per sample ranged from 1.55 to 17.3 million. Peptide binding affinities for the different alleles were predicted using the NetMHCIIPan4.1 algorithm, revealing allele‐specific binding imbalances. [file TAN-107-e70563-s004.docx]

| **Sample** | **Nº DCs (x10^6^)** | **Total peptides** | **Non-contaminant peptides** | **Binder peptides** | **Dual peptides** | **Peptides associated with each HLA-DRB1 allele** | | **Dual SB peptides** | **Dual WB peptides** |
| --- | --- | --- | --- | --- | --- | --- | --- | --- | --- |
| **P-DC1901** | 5.1 | 373 | 372 | 211 | 40 | DRB1*03:01 | 198 | 31 | 9 |
|  |  |  |  |  |  | DRB1*13:01 | 52 | 12 | 28 |
| **P-DC1902** | 8 | 160 | 158 | 58 | 12 | DRB1*13:05 | 33 | 3 | 9 |
|  |  |  |  |  |  | DRB1*15:01 | 37 | 1 | 11 |
| **P-DC1903** | 17.3 | 594 | 589 | 476 | 128 | DRB1*04:04 | 435 | 105 | 23 |
|  |  |  |  |  |  | DRB1*13:02 | 163 | 34 | 94 |
| **P-DC1907** | 6.1 | 284 | 280 | 232 | 55 | DRB1*07:01 | 156 | 44 | 11 |
|  |  |  |  |  |  | DRB1*13:02 | 131 | 13 | 42 |
| **P-DC1912** | 5.1 | 355 | 340 | 240 | 111 | DRB1*03:01 | 158 | 48 | 63 |
|  |  |  |  |  |  | DRB1*13:03 | 187 | 68 | 43 |
| **P-DC1914** | 3.4 | 124 | 120 | 59 | 20 | DRB1*01:02 | 52 | 14 | 6 |
|  |  |  |  |  |  | DRB1*13:02 | 27 | 6 | 14 |
| **C-DC1921** | 1.6 | 173 | 170 | 106 | 29 | DRB1*13:01 | 33 | 7 | 22 |
|  |  |  |  |  |  | DRB1*13:02 | 102 | 24 | 5 |
| **C-DC1922** | 5.4 | 83 | 82 | 53 | - | DRB1*07:01 | 53 | - | - |
| **C-DC2102** | 6.6 | 131 | 131 | 85 | 17 | DRB1*01:01 | 81 | 16 | 1 |
|  |  |  |  |  |  | DRB1*11:01 | 21 | 2 | 15 |
| **C-DC2103** | 7.6 | 909 | 894 | 491 | 69 | DRB1*11:04 | 191 | 47 | 22 |
|  |  |  |  |  |  | DRB1*12:01 | 369 | 14 | 55 |
| **C-DC2104** | 5 | 156 | 154 | 114 | 71 | DRB1*03:01 | 84 | 30 | 41 |
|  |  |  |  |  |  | DRB1*13:03 | 101 | 32 | 39 |
| **C-DC2105** | 4 | 217 | 217 | 138 | 74 | DRB1*01:01 | 109 | 61 | 13 |
|  |  |  |  |  |  | DRB1*07:01 | 103 | 34 | 40 |
| **C-DC2106** | 3.9 | 374 | 374 | 294 | 141 | DRB1*01:01 | 277 | 96 | 45 |
|  |  |  |  |  |  | DRB1*07:01 | 158 | 74 | 67 |
| **C-DC2107** | 4.9 | 98 | 96 | 52 | 26 | DRB1*03:01 | 41 | 14 | 12 |
|  |  |  |  |  |  | DRB1*13:02 | 37 | 17 | 9 |
| **Total** |  | 2,899 | 2,863 | 1,947 |  |  |  |  |  |

**Supplementary Table 1. Summary table showing the number of total, filtered, binder, and dual peptides identified in each sample, along with the number of peptides associated with each HLA-DRB1 allele.** The number of MoDCs obtained per sample ranged from 1.55 to 17.3 million. Peptide binding affinities for the different alleles were predicted using the NetMHCIIPan4.1 algorithm, revealing allele-specific binding imbalances.
