# Supplementary material for: HLA‐DRB1 Allelic Combinations Differentially Shape Dendritic Cell Antigen Presentation Enhanced by Tumour Cell Line Lysate‐Pulsing
Source: HLA. 2026 Jan 28;107(2):e70563. doi: 10.1111/tan.70563 (PMC12853012; doi:10.1111/tan.70563)
Supplement: Supplementary file 5 — Table S4: Summary table showing the number of pulse‐derived peptides and proteins associated with each HLA‐DRB1 allele in every sample. P‐DC1902 was excluded as no pulse‐derived proteins were identified. [file TAN-107-e70563-s006.docx]

| **Sample** | **Peptides-source pulse-derived proteins with affinity for HLA-DRB1 alleles** | **HLA-DRB1 allele** | **Peptides-proteins associated with each HLA-DRB1 allele** |
| --- | --- | --- | --- |
| **P-DC1901** | 18 - 9 | DRB1*03:01 | 17-8 |
|  |  | DRB1*13:01 | 2-2 |
| **P-DC1903** | 84 - 39 | DRB1*04:04 | 80-37 |
|  |  | DRB1*13:02 | 25-14 |
| **P-DC1907** | 8 - 4 | DRB1*07:01 | 3-2 |
|  |  | DRB1*13:02 | 7-4 |
| **P-DC1912** | 12 - 11 | DRB1*03:01 | 9-7 |
|  |  | DRB1*13:03 | 8-7 |
| **P-DC1914** | 6 - 6 | DRB1*01:02 | 6-6 |
|  |  | DRB1*13:02 | 3-3 |
| **Total** | 120 - 58 |  |  |

**Supplementary Table 4. Summary table showing the number of pulse-derived peptides and proteins associated with each HLA-DRB1 allele in every sample.** P-DC1902 was excluded as no pulse-derived proteins were identified.
